# Quantitative proteomic analysis of the influence of lignin on biofuel production by *Clostridium acetobutylicum* ATCC 824

**DOI:** 10.1186/s13068-016-0523-0

**Published:** 2016-05-31

**Authors:** Mahendra P. Raut, Narciso Couto, Trong K. Pham, Caroline Evans, Josselin Noirel, Phillip C. Wright

**Affiliations:** The ChELSI Institute, Department of Chemical and Biological Engineering, University of Sheffield, Mappin Street, Sheffield, S1 3JD UK; Chaire de Bioinformatique, LGBA, Conservatoire National Des Arts Et Métiers, 75003 Paris, France; School of Chemical Engineering and Advanced Materials, Faculty of Science, Agriculture & Engineering, Newcastle University, Newcastle upon Tyne, NE1 7RU UK

**Keywords:** *Clostridium acetobutylicum*, Cellobiose, Lignin, Fermentative end products, Biofuel production, Quantitative proteomics, iTRAQ, Metabolic changes

## Abstract

**Background:**

*Clostridium acetobutylicum* has been a focus of research because of its ability to produce high-value compounds that can be used as biofuels. Lignocellulose is a promising feedstock, but the lignin–cellulose–hemicellulose biomass complex requires chemical pre-treatment to yield fermentable saccharides, including cellulose-derived cellobiose, prior to bioproduction of acetone–butanol–ethanol (ABE) and hydrogen. Fermentation capability is limited by lignin and thus process optimization requires knowledge of lignin inhibition. The effects of lignin on cellular metabolism were evaluated for *C. acetobutylicum* grown on medium containing either cellobiose only or cellobiose plus lignin. Microscopy, gas chromatography and 8-plex iTRAQ-based quantitative proteomic technologies were applied to interrogate the effect of lignin on cellular morphology, fermentation and the proteome.

**Results:**

Our results demonstrate that *C. acetobutylicum* has reduced performance for solvent production when lignin is present in the medium. Medium supplemented with 1 g L^−1^ of lignin led to delay and decreased solvents production (ethanol; 0.47 g L^−1^ for cellobiose and 0.27 g L^−1^ for cellobiose plus lignin and butanol; 0.13 g L^−1^ for cellobiose and 0.04 g L^−1^ for cellobiose plus lignin) at 20 and 48 h, respectively, resulting in the accumulation of acetic acid and butyric acid. Of 583 identified proteins (FDR < 1 %), 328 proteins were quantified with at least two unique peptides. Up- or down-regulation of protein expression was determined by comparison of exponential and stationary phases of cellobiose in the presence and absence of lignin. Of relevance, glycolysis and fermentative pathways were mostly down-regulated, during exponential and stationary growth phases in presence of lignin. Moreover, proteins involved in DNA repair, transcription/translation and GTP/ATP-dependent activities were also significantly affected and these changes were associated with altered cell morphology.

**Conclusions:**

This is the first comprehensive analysis of the cellular responses of *C. acetobutylicum* to lignin at metabolic and physiological levels. These data will enable targeted metabolic engineering strategies to optimize biofuel production from biomass by overcoming limitations imposed by the presence of lignin.

**Electronic supplementary material:**

The online version of this article (doi:10.1186/s13068-016-0523-0) contains supplementary material, which is available to authorized users.

## Background

Due to growing uncertainties regarding the supply and cost of fuel transportation and concerns about their related environmental impact, the sustainable production of clean energy has become a strategic priority. Lignocellulosic biomass has great potential as a prime feedstock for future biofuel generation, since it is promising source of mixed sugars for fermentative biofuels and chemical production thereby ensuring renewable and sustainable source of energy and also reducing environmental impacts [1]. For that reason, anaerobic *Clostridia* have received much attention in recent years because of their ability to produce alternative biofuels from renewable biomass and agricultural waste materials [2]. In particular, *Clostridium acetobutylicum* ATCC 824 (*C. acetobutylicum*) is a promising candidate. Although it is unable to utilize biopolymers (cellulose and hemicellulose) directly, [3] it can ferment wide range of biomass saccharides (such as cellobiose) into acetone–butanol–ethanol (ABE) [2] products and hydrogen (H_2_) [4] once brought into solution (hydrolysate) by pre-treatment. Since the presence of lignin [forming 20–30 % of lignocellulosic (wood) biomass] is a key challenge in biological/enzymatic hydrolysis of lignocellulose, [5, 6] various chemical pre-treatments are widely employed to obtained biomass hydrolysates (pre-treatment liquor).

However, major bottlenecks still hamper the economics of ABE production from biomass hydrolysate; in particular the production of lignin and its derivatives during chemical pre-treatments which have inhibitory effects on *Clostridium* on biofuel production [7]. The phenolic compounds from lignin degradation have been demonstrated as the main inhibitor of ABE fermentation by *Clostridia* [2, 8]. Alkali treatments at high temperature and pressure have been shown to be most effective technique for biomass pre-treatment to release fermentable sugars and most of the dissolved native lignins into the pre-treatment liquor [9]. Understanding the effects of lignin alone on *Clostridium* biology, with particular focus on ABE production, is key to mimic such hydrolysates and process optimization to target improved yield.

This study combines an analysis of the effect of lignin on cellobiose consumption, growth rate, morphology, ABE production with a quantitative proteomic analysis to measure alterations in proteins associated with the ‘lignin bottleneck’. A soluble form of Kraft lignin, i.e. alkali lignin (carboxylated), was selected since previous studies into microbial degradation of lignin and bioconversion to value-added products have used Kraft lignins [10–15]. Since, metabolism in *C. acetobutylicum* is biphasic, with acidogenesis (acetic acid, butyric acid and H_2_) dominant during the exponential phase and solventogenesis (ABE) dominant during stationary phase [16], the proteome was relatively compared at specific time points (exponential and stationary phases) during growth on either cellobiose or cellobiose supplemented with lignin. This study employed 8-plex isobaric tags for relative and absolute quantitation (iTRAQ) to quantitatively profile biological replicates of the four sample types. Data were integrated with gas chromatographic (GC) analysis of ABE and H_2_ production.

## Results and discussion

**Fig. 1:**
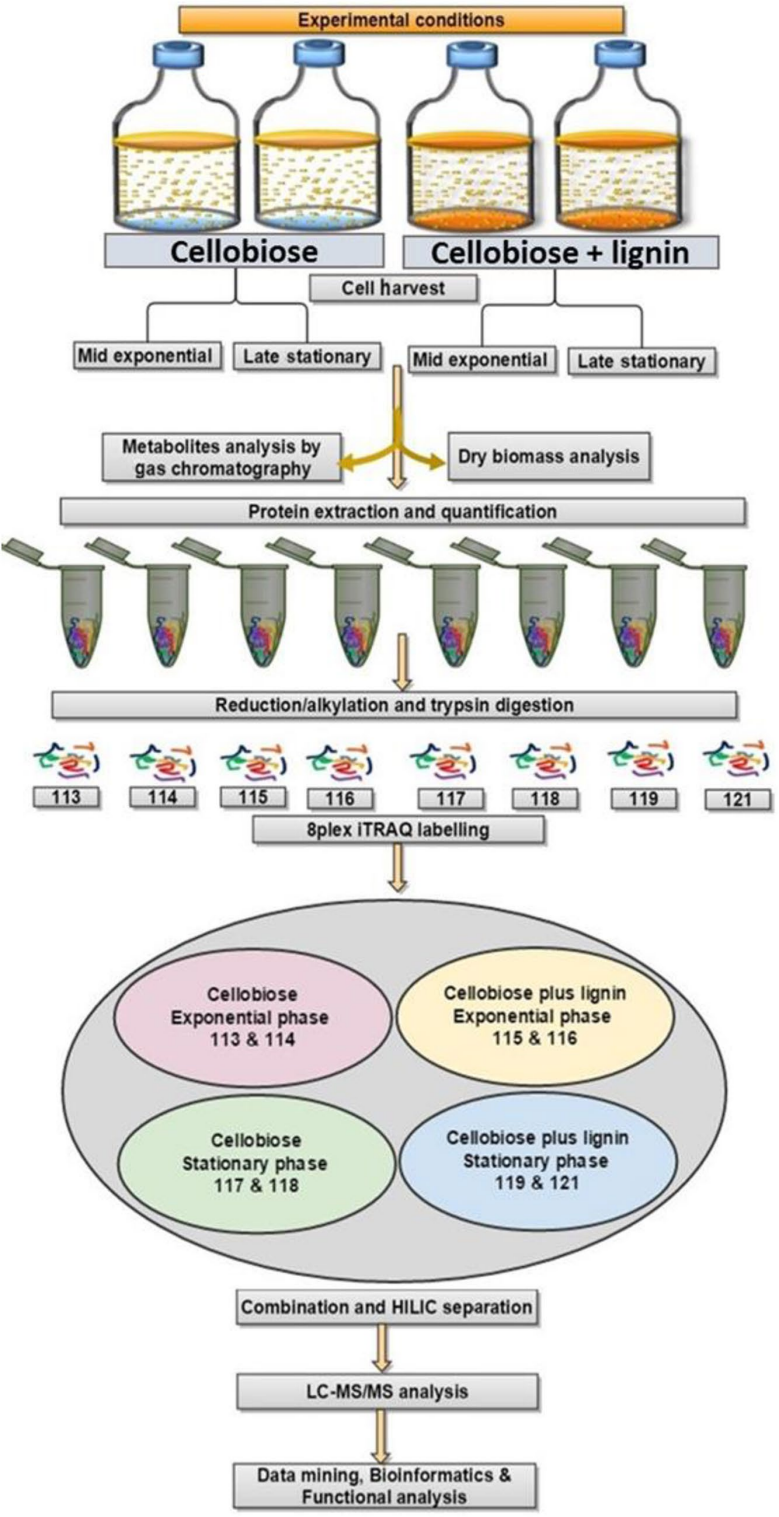
8-plex iTRAQ proteomic workflow. Proteins from eight individual samples (4 each for C and CL 2, exponential and 2 stationary phases) were digested into peptides that were tagged with isobaric stable isotope-labelled reagents. Relative quantification information was extracted upon collision-induced dissociation. 8-plex iTRAQ reagents tags have eight unique reporter ions of specific mass-to-charge (*m/z*) values (113, 114, 115, 116, 117, 118, 119 and 121) that produced peptide fragmentation during tandem MS and are used for relative quantitation by relative peak intensity. Fragmented peptide ions were used for peptide ID and protein identification. Samples for hydrogen, metabolites and dry cell biomass measures were taken in parallel

Carbohydrate polymers (cellulose and hemicellulose) and aromatic polymers (lignin) are the major components of lignocellulosic biomass that, upon hydrolysis (alkali/acid or enzymatic), produces fermentable sugars (that can be utilized by *C. acetobutylicum*) and non-fermentable phenol compounds. Therefore, it is very important to understand how the presence of lignin affects fermentation end products (ABE) formation and core metabolic pathways. This study focused on metabolic and physiological changes in *C. acetobutylicum* during growth on cellobiose only (hereafter: C condition) and cellobiose plus lignin (hereafter: CL condition) supplemented conditions. The workflow shown in Fig. 1 demonstrates the integrated metabolic and proteomics analysis.

### Effect of lignin on the cell morphology, growth and fermentative end products

**Fig. 2:**
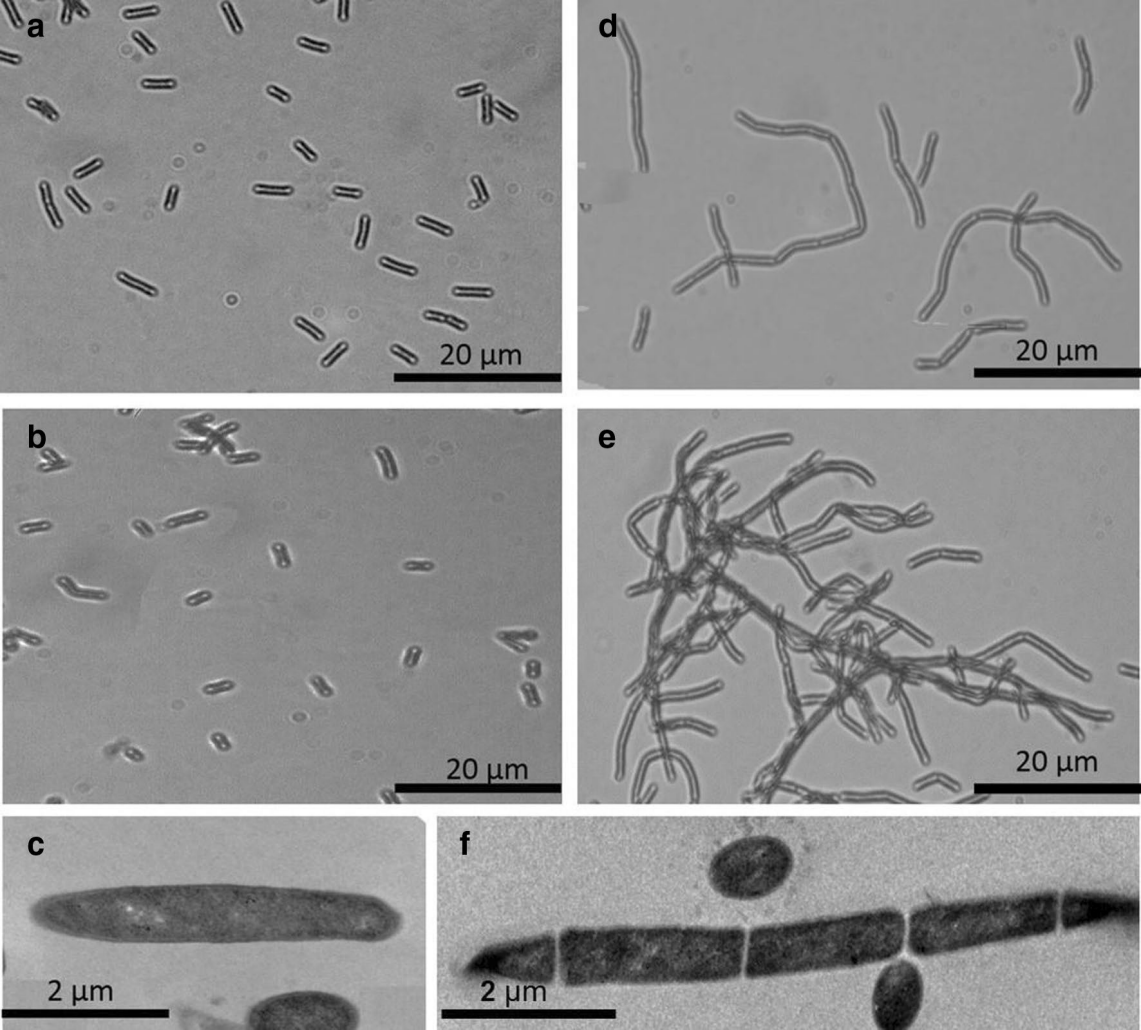
Morphology of *C. acetobutylicum* cells grown on C (**a**–**c**) and CL (**d**–**f**) at exponential phase (16 h) as shown in **a** and **d** and stationary phase (48 h) as shown in **b** and **e**. **a**–**d** were obtained by Olympus BX51 microscopy at ×60 magnification and **c** and **e** were obtained by transmission electron microscopy (TEM) at ×11,000 magnification

**Fig. 3:**
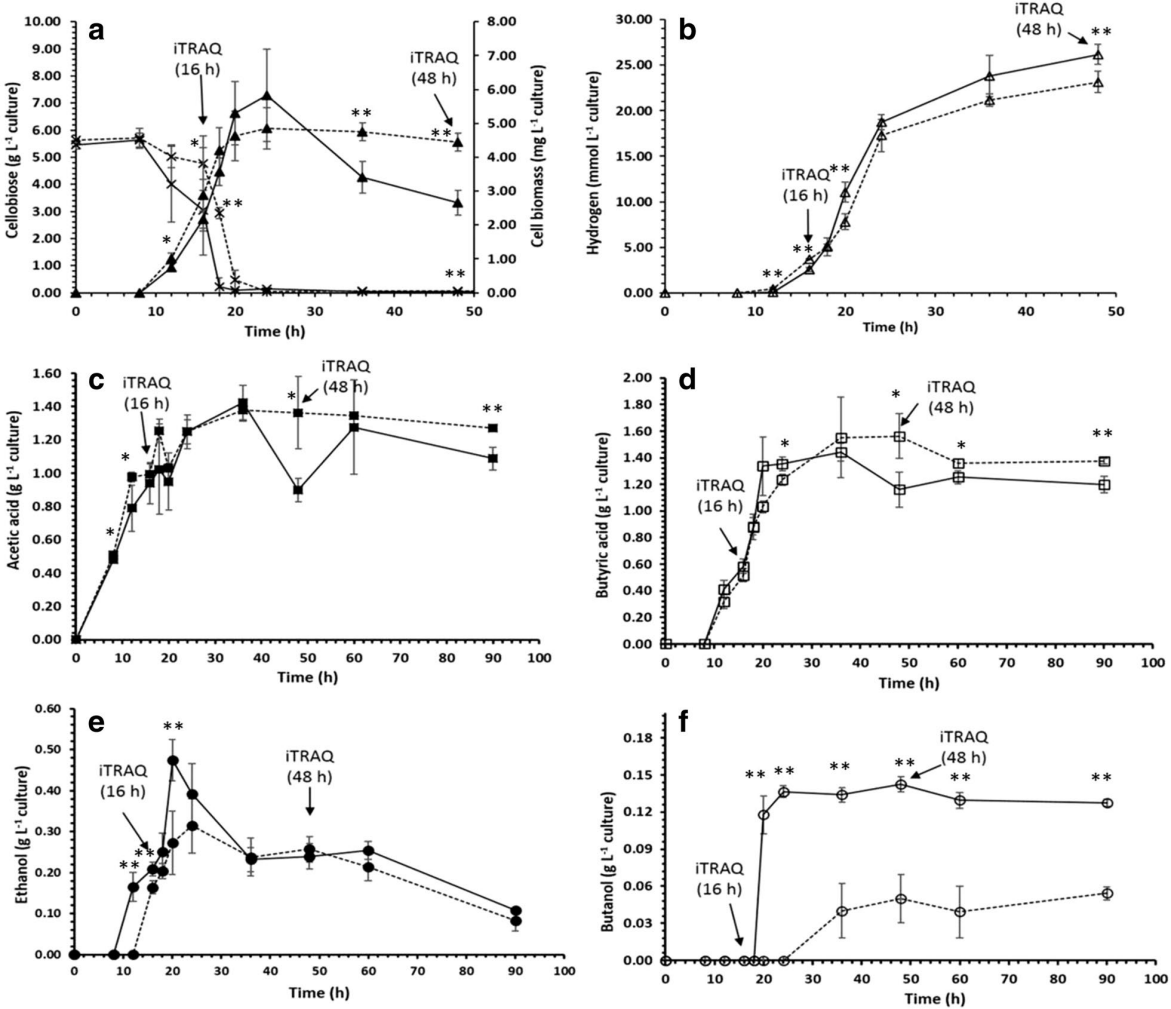
Growth, cellobiose consumption and metabolite formation during fermentation in *C. acetobutylicum* under CL (*dashed line*) and C (*solid line*) conditions; **a** Growth/dry cell biomass (*closed triangle*), cellobiose consumption profiles (*striked cross*), **b** H_2_ production (*open triangle*), **c** acetic acid (*closed square*), **d** butyric acid (*open square*) and **e** ethanol (*closed circle*) and **f** butanol (*open circle*). Data were taken from four biological replicates and mean values with the *error bars* indicate standard error of the mean. *Arrows* indicate the sampling points for iTRAQ quantitative proteomic analysis (16 and 48 h). *p ≤ 0.05 and **p ≤ 0.0099

Microscopic observations showed filamentous morphology of *C. acetobutylicum* with asymmetric and phenotypic cell division when lignin was present in combination with cellobiose (Fig. 2d–f) relative to cellobiose alone (Fig. 2a–c) in both exponential and stationary phases. The morphological changes suggest that the presence of lignin challenged the bacterial metabolism but did not affect the growth (based on dry cell biomass as shown in Fig. 3a), since similar growth trends were observed until the late exponential phase (16 h) for both C and CL. Upon reaching stationary phase, a significant reduction in cell biomass was observed in C compared to CL at 36 h (Fig. 3a). Alteration in cell morphology is a visible indicator of bacterial adaptation strategies to tackle different environmental stress conditions [17]. Reduction in cell biomass concentration in the C condition could reflect reduced cell size and sporulation during the stationary phase (Fig. 2a, b versus Fig. 2d, e) as previously proposed by Steiner et al. [18]. A comparatively faster rate of cellobiose consumption was observed in C media compared to CL media in the exponential phase (Fig. 2a), consistent with lignin inhibition.

Metabolites production was also validated: acetic acid and butyric acid and hydrogen (H_2_); acetone, ethanol and butanol were detected and quantified by GC, to measure acidogenesis and solventogenesis, respectively. Metabolite profiles between C and CL are shown in Fig. 3b–f (Additional file 1: Metabolites data). Metabolite production was significantly affected by the presence of lignin. The major change was lignin inhibition of butanol production in both exponential and stationary phases (Fig. 3f). Ethanol production was inhibited by lignin during exponential phase, but not stationary phase (Fig. 3e). Acetone production was below the level of detection for both C and CL conditions. In terms of acidogenesis, hydrogen production was statistically significantly lower in the CL versus the C condition throughout the experiment (Fig. 3b). The onset of acetic acid production began as soon as cells started to grow (8 h) and reached a maximum at 36 h (Fig. 3c), which was followed by butyric acid production, starting at 12 h and reaching a maximum at 36 h (Fig. 3d). Afterwards, a statistically significant decrease in acetic acid and butyric acid concentrations (*p* values of 0.018 and 0.01, respectively) in C versus CL was observed that can be correlated with the statistically significantly higher ethanol and butanol production (*p* values of 0.006 and 0.0021, respectively) in the C condition versus CL (ethanol; 0.47 g L^−1^ for C and 0.27 g L^−1^ for CL and butanol; 0.13 g L^−1^ for C and 0.04 g L^−1^ for CL) (Fig. 3e, f). This indicates a rapid production of solvents in C versus CL and the accumulation of acids in the presence of lignin. Furthermore, there was delayed production of solvents in the presence of lignin, i.e. the onset of ethanol production started at 10 and 12 h for C and CL conditions and comparatively late butanol production started at 18 and 36 h for C and CL conditions, respectively. This is interesting that morphological changes (observed during transition of exponential to stationary) and reduced cell dry biomass concentration occur in C versus CL (Figs. 2, 3a).

In general, it is presumed that acid production and solvent production occur at different stages, but our results suggest that simultaneous acid and solvent production is occurring (metabolic shift) [19] during growth. Our results have some agreement though, with previous studies that show simultaneous production of acids and solvents during growth and suggest that acidogenic and solventogenic cells co-exist in the culture [2, 19].

Overall, the presence of lignin in the growth medium resulted in less H_2_ and solvent production (Fig. 3b, e, f). Usually, higher H_2_ production occurs when acetic acid and butyric acid are produced during the acidogenic growth phase (exponential phase), [4, 20] and it is believed to be due to accumulation of organic acids as a function of pH [21]. Acetic acid, butyric acid and H_2_ were reasonably high and concomitantly produced in both treatment conditions, which is in agreement with previous findings that a mixture of acetic acid and butyric acid as a fermentative product yields more H_2_ [22]. However, production seemed to be continued through the mid-stationary phase, indicating simultaneous acid and solvent production is possible, as previously observed [20].

Interestingly, acetone production was not observed. The results are consistent with previous observations in *C. acetobutylicum* when grown on cellobiose as the main carbon source, where higher H_2_, ethanol and acetic acid were produced as the main by-products and little or no acetone and butanol production was observed [23–25]. This study demonstrates substrate specificity and substrate-dependent fermentation flexibility of this bacterium.

Despite the versatility of *C. acetobutylicum* in producing acids, H_2_ and ABE, very little is known about the dynamic regulation of metabolic networks, stoichiometry and directionality of metabolic fluxes in this bacterium [26]. It is believed that the efficiency of substrate conversion to final product solely depends on the direction of carbon intermediates and electron flow in the fermentation pathway [27, 28]. Therefore, since lignin had a negative effect on growth, morphology and fermentative products of *C. acetobutylicum,* we investigated this further by iTRAQ-based proteomics to gain insight into differential regulation of key proteins in the presence of lignin by comparing the exponential and stationary phases of cells grown under C and CL conditions.

### Effect of lignin on *C. acetobutylicum* metabolism: an iTRAQ-based quantitative proteomic approach

**Fig. 4:**
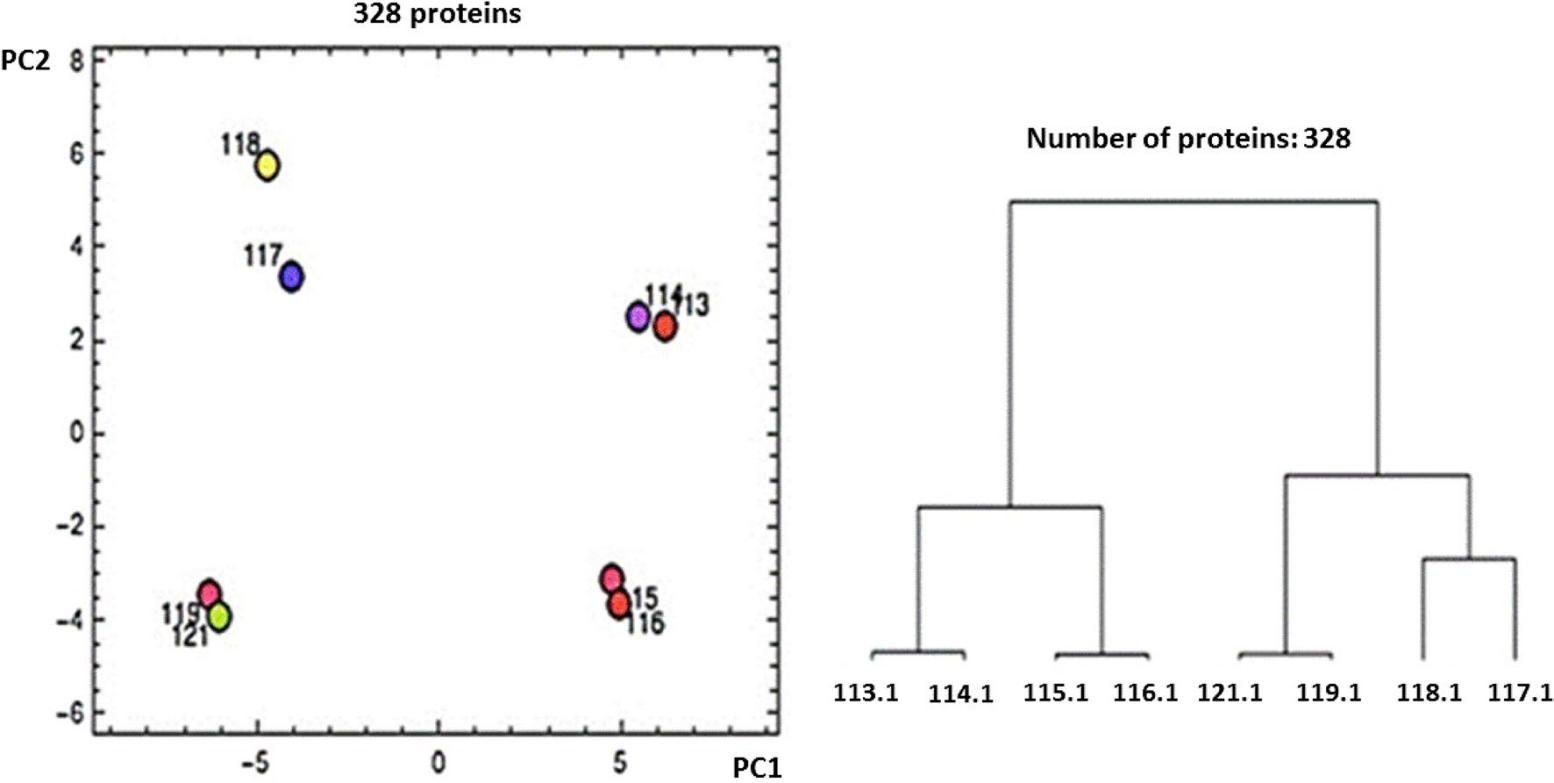
Hierarchical clustering (dendrogram) and principal component analysis (PCA) plot of proteome analysis data that characterizes the trend exhibited by the differentially expressed protein profile of ExpC (113,114), ExpCL (115,116), StaC (117,118) and StaCL (119,121)

A biological duplicate of each exponential and stationary phase from C and CL was chemically labelled using 8-plex iTRAQ reagents (following tryptic digestion to generate peptides) and analysed by HPLC–MS/MS analysis. Of 583 identified proteins (FDR < 1 %), 328 proteins were quantified with at least two unique peptides [29] (Additional file 2: Proteomic data). To evaluate replicates, hierarchical clustering (dendrogram) and principal component analysis (PCA) were applied to the iTRAQ reporter ion intensities. Hierarchical clustering (dendrogram form) and PCA analyses revealed that there was a clear clustering and distinction between biological replicates. Moreover, the proteomes of C and CL grown cells in their respective exponential and stationary phases were clustered closely in the same size of the dendrogram and PCA (Fig. 4) indicating similarity between replicates and differences between biological conditions. Notably, the proteome in all four comparisons (each of exponential and stationary phase of C and CL condition) was evidently different as shown in Fig. 4.

The effects of various lignin derivative compounds, metabolites and substrates stresses on *Clostridia* have been recently reviewed by Baral and Shah [7]. In particular, in *C. acetobutylicum,* several transcriptomics studies were dissected to study phase-related metabolism [30], physiological changes [31] and butanol stress or tolerance [32], but very few studies have been performed at the quantitative proteomics level. In a rare example, an adaptive stress response of *C. acetobutylicum* to the toxic metabolites, butyrate and butanol, was recently analysed by Vekantraman et al. [33] using iTRAQ-based quantitative proteomics.

**Fig. 5:**
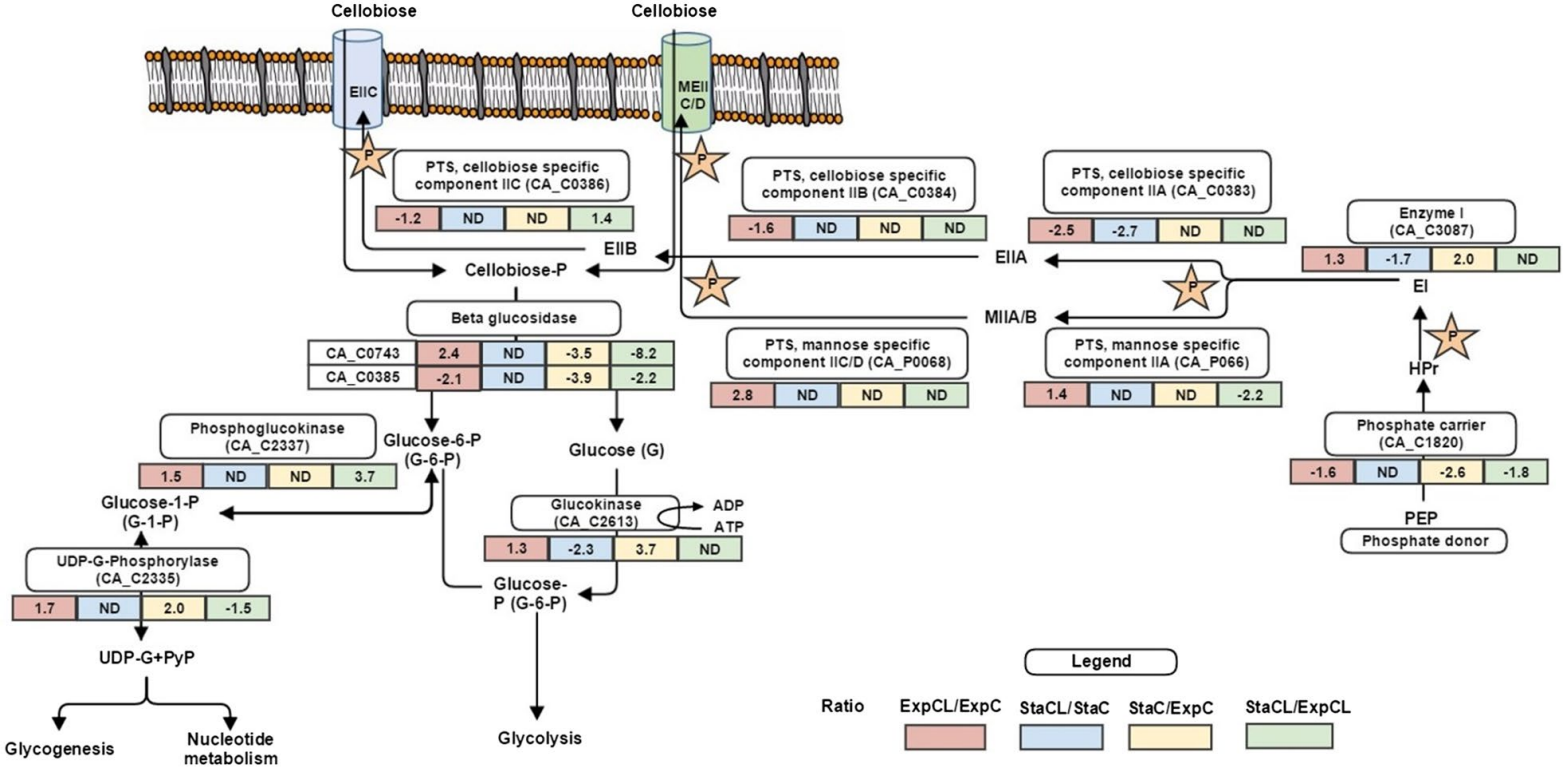
Alterations in relative abundance of protein expressions (iTRAQ ratio representing fold changes) in sugar transportation during growth on C and CL. [Comparing ratio; ExpCL/ExpC (*red*), StaCL/StaC (*blue*), StaC/ExpC (*yellow*) and StaCL/ExpCL (*green*)]. (Fold change in protein expression: *negative values* indicate reduced abundance of proteins and positive values indicate increased abundance of proteins). The *stars* indicate the phosphoryl group of PEP, which is transferred to the imported cellobiose via series of PTS system proteins

**Fig. 6:**
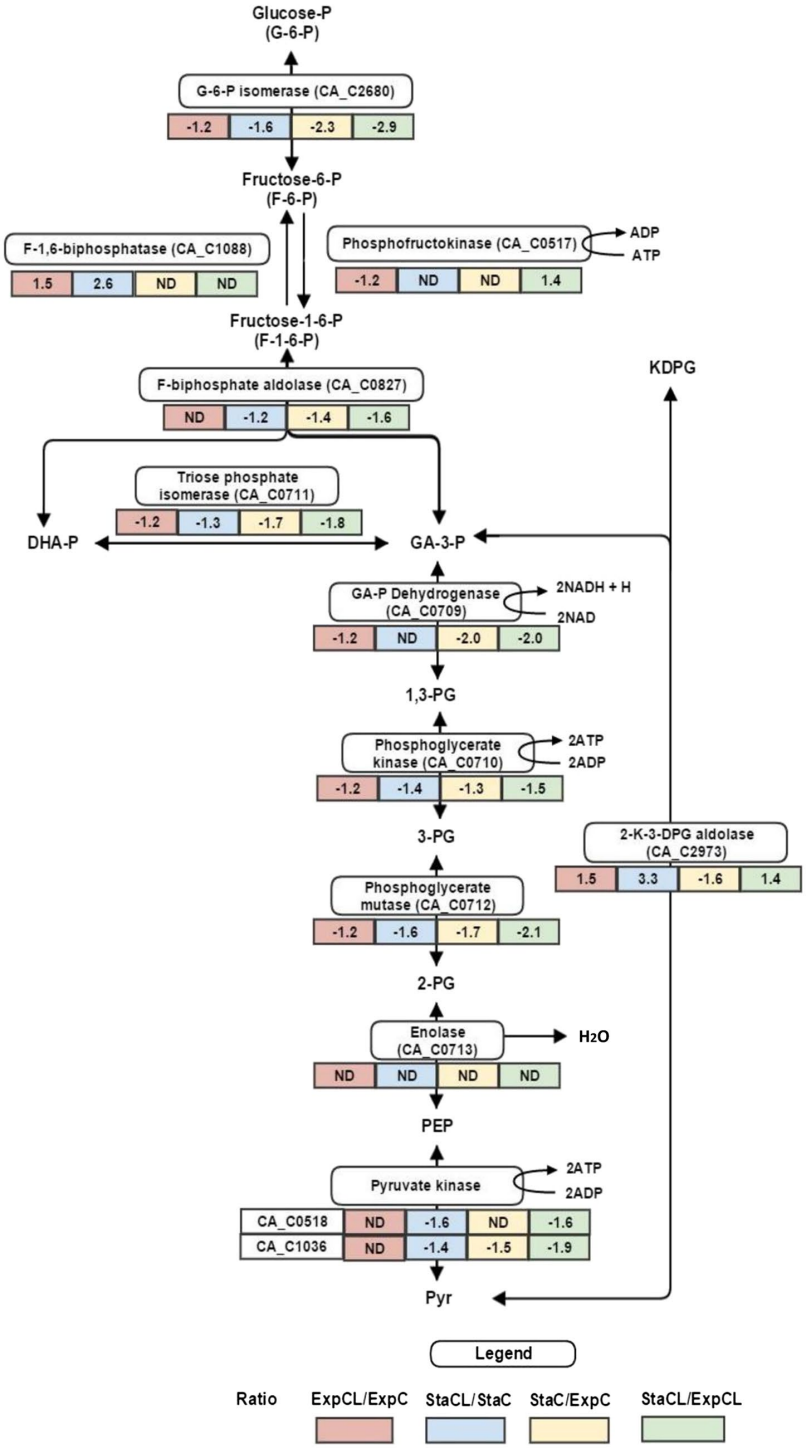
Alterations in relative abundance of protein expressions (iTRAQ ratio representing fold changes) in glycolysis during growth on C and CL. [Comparing ratio; ExpCL/ExpC (*red*), StaCL/StaC (*blue*), StaC/ExpC (*yellow*) and StaCL/ExpCL (*green*)] (Fold change in protein expression: *negative values* indicate reduced abundance of proteins and positive values indicate increased abundance of proteins)

**Fig. 7:**
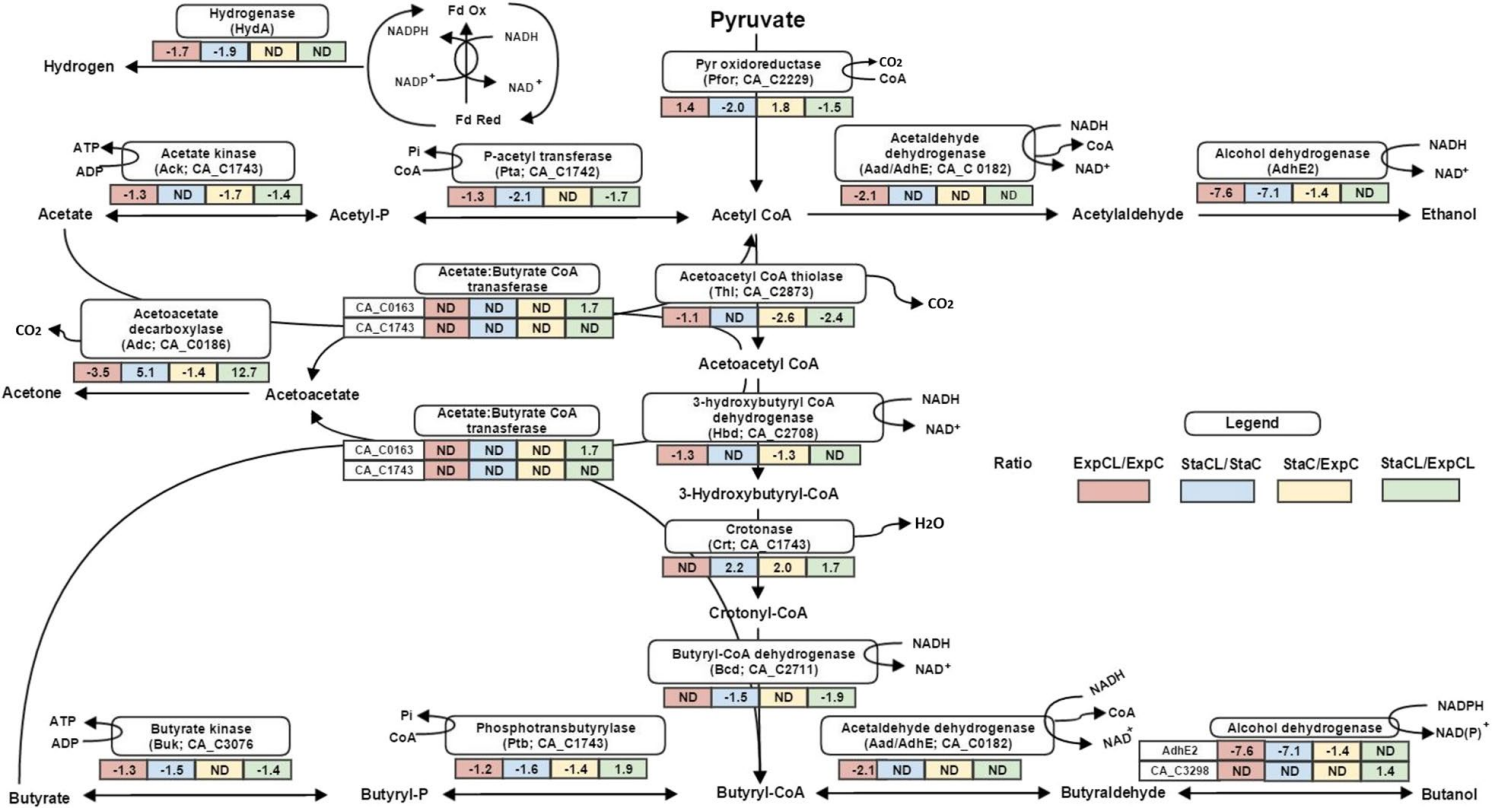
Alterations in relative abundance of protein expressions (iTRAQ ratio representing fold changes) during pyruvate to end products formation during fermentation in *C. acetobutylicum.* The *diagram* represents protein profiling changes during lignin stress conditions in *C. acetobutylicum* (Fold change in protein expression: *negative values* indicate reduced abundance of proteins and positive values indicate increased abundance of proteins). Comparing ratio; ExpCL/ExpC (*red*), StaCL/StaC (*blue*), StaC/ExpC (*yellow*) and StaCL/ExpCL (*green*)

Our proteomics data provide vital information on differential expressions of proteins, thus providing a more detailed understanding of the effect of lignin on various cellular functions in *C. acetobutylicum*. A total 158 and 134 proteins were found to be differentially regulated in the exponential phase and stationary phases of CL, respectively, when compared to the exponential and stationary phases of cells grown in C media (ExpCL/ExpC and StaCL/StaC). Moreover, changes in protein expressions were also observed when shifting from exponential to stationary phase occurring in their respective conditions. In total, 173 and 216 proteins were differentially expressed in C (StaC/ExpC) and CL (StaCL/ExpCL) conditions, respectively. These significantly differentially expressed proteins were mapped into pathways and the results are summarized in Figs. 5, 6 and 7. Lignin significantly changed the cellular functions of *C. acetobutylicum*, namely sugar transport, glycolysis, fermentative pathways, DNA replication, transcription/translation, cell division, sporulation/stress response and cell signalling/secretion. Therefore, these pathways/enzymes are discussed and correlated individually in the following sections.

### Protein expression changes associated with cellobiose transport and glycolysis regulation

Our iTRAQ proteomics results revealed that proteins involved in the cellobiose transport system and glycolytic pathway were altered in expression levels by the presence of lignin. The majority of enzymes in these pathways were down-regulated in both exponential and stationary phase in the presence of lignin relative to the cellobiose-only condition.

*Clostridium acetobutylicum* can utilize a wide variety of carbohydrate sugars as carbon source and has multiple adaptable sugar transport and metabolic processes that are specifically regulated at the transcriptional level depending on environmental stress and nutrient conditions [34]. The phosphoenolpyruvate (PEP)-linked phosphotransferase system (PTS) is the major sugar transportation system exhibited in *C. acetobutylicum.* The PTS-dependent sugar transport systems are shown in Fig. 5. Usually, *C. acetobutylicum* uses a cellobiose-specific PTS system for cellobiose phosphorylation and transportation. The system consists of four proteins: PTS IIA (CA_C0383), PTS IIB (CA_C0384) and PTS IIC (CA_C0386), and β glucosidase (CA_C0385). In this study, the cellobiose-specific PTS complex was down-regulated in the presence of lignin (ExpCL/ExpC). However, the data suggest that to maintain the sugar level, cells adaptively switched to the non-specific mannose PTS system. The components of the non-specific mannose PTS system are IIA (CA_P0066), IIC/D (CA_P0068) and 6-β-glucosidase (CA_C0743) (possibly associated with mannose PTS system). These were found to be up-regulated (ExpCL/ExpC). The data show that *C. acetobutylicum* possesses multiple cellobiose transport systems, as suggested by Servinesky et al. [34]. Thus, adaptive activation of different transport systems in different environmental stress conditions could be possible and reflected at the level of the proteome, which represents a novel finding.

The cellobiose/mannose PTS systems allow cellobiose to enter the cell, so that it can be further cleaved into glucose and glucose 6-P by β-glucosidases and enter the glycolytic pathway. Non-phosphorylated glucose molecules are further phosphorylated by glucokinase (CA_C2613) to glucose-6-P; a protein was found to be in increased abundance in CL (ExpCL/ExpC). Glucose-6-P then enters into glycolysis (Fig. 6). In *C. acetobutylicum*, glucose to pyruvate conversion generally occurs via the Embden–Meyerhof–Parnas (EMP) pathway; the majority of measured proteins of the EMP pathway were down-regulated in the presence of lignin in the exponential phase. Since the metabolism of sugar presumably takes place during the exponential phase of *C. acetobutylicum*, the ratio of ExpCL/ExpC was considered to be crucial for the changes in the glycolysis process. Remarkably, the adaptive evolutionary enzyme 2-keto-3-deoxy-6-phosphogluconate aldolase (CA_C2973) was observed to be significantly up-regulated in the presence of lignin in both exponential and stationary phases; this enzyme can reversibly catalyse KDPG to pyruvate and glyceraldehyde-3-phosphate, bypassing the entire glycolytic process without any ATP production [35–37]. This protein is a vital protein in the modified Entner–Doudoroff (ED) pathway found in Archaea and *Clostridium aceticum* growing in extreme conditions [38]. Microbes using different glucose catabolic pathways is a little known fact; however, it solely depends on species and culture conditions [39]. Our results show that in presence of lignin, where EMP pathway proteins were down-regulated, cells might have attempted to maintain the cellular pyruvate/acetyl CoA level to achieve normal metabolic functions. This protein skips crucial steps in glycolysis, resulting in no net ATP generation. Although enolase (CA_C0713) was identified in this study, no differential expression was found. Our data show a reduced abundance of phosphofructokinase (CA_C0517) and this was adaptively accompanied by a reverse reaction catalysed by protein fructose 1,6-biphosphatase (CA_C1088), which had an elevated abundance in the lignin stress condition. Interestingly, tricarboxylic acid (TCA) cycle proteins aconitase (CA_C0971) and NAD-isocitrate dehydrogenase (CA_C0972) were up-regulated in the exponential phase of CL grown cells (ExpCL/ExpC). These proteins are involved in energy harvesting via NADH/NADPH generation [40]. Therefore, it can be possible that to compensate for low NADH derived from glycolysis in the presence of lignin, the bacterium adaptively increased expression of these proteins.

### Protein expression changes associated with fermentation pathway regulation

*Clostridium acetobutylicum* is a model organism for ABE fermentation. The enzymes involved in acid and solvent production pathways were identified in both conditions with differential expressions. In agreement with the negative effect of lignin on glycolysis, the subsequent fermentation pathways were also found to be down-regulated in CL conditions. The network of differentially expressed proteins during acidogenesis and solventogenesis is shown in Fig. 7.

The major route for the synthesis of fermentation products starts with pyruvate, which is first converted into acetyl CoA, carbon dioxide (CO_2_) and reduced ferredoxin (or flavodoxin; Fld) [41]. This further undergoes a biphasic branched fermentation process, where acids (formic acid, acetic acid and butyric acid) are produced during acidogenesis and solvents (ethanol and butanol) are produced during solventogenesis [42, 43]. Reduced flavodoxin (Rd) is further used as an electron carrier for either NADH, NAD(P)H or hydrogen production depending on the cellular states [44]. In this study, we found increased acetic acid and hydrogen production, indicating carbon and electron flow towards the molecular hydrogen production and carbon flow towards acetic acid in both conditions. The hydrogenase (HydA) that receives electrons from flavodoxin (Fed) and produces hydrogen was only quantified by a single unique peptide at low abundance in CL conditions. Even though tentative, this observation is in agreement with lower hydrogen production in the CL condition (Fig. 3b). The enzymes involved in acetic acid and butyric acid production were down-regulated in lignin condition (CL), particularly, acetate kinase (CA_C1743), phosphate acetyltransferase (CA_C1742), acetaldehyde dehydrogenase (CA_P0162), butyrate kinase (CA_C3075) and phosphate transbutyrylase (CA_C3076) (Fig. 7). However, no significant difference was observed in acids production in both conditions, but Fig. 3c, d indicates decreased acid production during the stationary phase of C, this possibly explains the utilization of acids for solvent production (ethanol and butanol) was faster in C than CL conditions. This can be further justified by subsequent down-regulation of solvent-producing enzymes in CL: acetaldehyde dehydrogenase (CA_C0162) and (AdhE2) (ExpCL/ExpC and StaCL/ExpC). Down-regulation of these enzymes suggests less conversion of acids into solvents in CL. This is in agreement with our metabolite analysis (Fig. 3e, f). Interestingly, the protein acetoacetate decarboxylase (CA_P0165) (involved in acetone production) was differentially regulated across all comparisons, but no acetone production was observed during fermentation. No expression of acetate/butyrate CoA transferase (CA_P0163, CA_P0164) was observed in both conditions, indicating that these enzymes possibly controlled acetone production in *C. acetobutylicum*. Providing evidence to this suggestion, it was previously found that acetone production was controlled at the transcriptional level through the expression of CoA transferase, but not through the expression of acetoacetate carboxylase [44, 45]. In addition, our results are in agreement with previous studies that suggested that higher hydrogen production results in less or no acetone production when *C. acetobutylicum* was grown on cellobiose as the substrate [23–25]. Some studies also found that higher hydrogen partial pressure resulted in lower acetone production [27, 46]. As seen in our data, two major ATP generation pathways, i.e. glycolysis and acid production were down-regulated in lignin conditions, likely resulting in lower ATP production. This could be the possible reason for reduced abundance of those proteins that possess ATP-dependant activity with a consequent effect on various functions related to transcription/translation and in cell division/sporulation.

### Protein expression changes associated with DNA metabolism/transcription/translation

Since *C. acetobutylicum* produces acids and solvents at different stages of its life cycle and lignin affects their production due to changes in metabolism as confirmed by GC and proteomic data, we decided to investigate other metabolic functions (e.g. DNA metabolism, transcription/translation) that may contribute to this behaviour and also correlated to changes in cell morphology. Fold changes in protein expression levels of various other metabolic functions during growth on C and CL are shown in Additional file 3: Fig. S1 and Table S1. These changes appear to manifest as an adaptive survival strategy of this bacterium at the replication, transcription and translation level [47]. The regulation of the most relevant proteins to lignin-induced changes is discussed in the following sections.

The proteins involved in DNA repair, maintenance and stabilization that were only found in CL (StaCL/ExpCL), including DNA-binding protein HU (CA_C3211), nucleoid-associated protein (CA_C0126), and single-stranded DNA-binding protein (CA_C2382). These proteins were up-regulated in the presence of lignin, suggesting that DNA damage was induced by lignin, thus the repair system worked efficiently.

We found that many transcriptional proteins were observed to be present in low abundance when comparing stationary phases to their respective exponential phases in C and CL conditions. However, the adaptive transcriptional regulator Lrp family protein (CA_C0977) [47] and GTP sensing transcriptional pleiotropic repressor CodY protein (CA_C1786) [48] were up-regulated in CL conditions (ExpCL/ExpC and StaCL/StaC). GTP binding CodY protein suppresses many genes of transition from exponential to the stationary/sporulation phase by binding to DNA [48, 49]. Increased abundance of this protein in CL conditions may correlate with a delay in sporulation/defective cell division in CL conditions. In addition, the transcription regulatory septation protein SpoVG (CA_C3223) (involved in a site-specific DNA-binding activity) [50] was down-regulated in the presence of lignin and, therefore, cells may not have been able to control normal cell division and sporulation conditions, resulting in the filamentous morphology observed with CL grown cells.

We also identified 33 ribosomal proteins differentially regulated between the CL and C conditions. However, it was observed that during the exponential phase of CL conditions, most of the ribosomal proteins were increased in abundance (ExpCL/ExpC). These proteins were also increased in abundance during stationary phases compared to their respective exponential phases (StaC/ExpC and StaCL/ExpCL). These proteins are normally down-regulated during stress conditions and stationary phases. The possible reason for increased abundance of these proteins could be correlated to the low expression of the ATP-dependent Lon protease (CA_C2637), which degrades ribosomal proteins when cells starve for amino acids [51]. This effect could be possible, since a major translational regulator [52] protein TYPA/BIPA ATPase (CA_C1684) was significantly down-regulated in CL conditions. This protein alters ribosomal structure/function to achieve normal translation in *C. acetobutylicum.* The activity of this protein has been shown to be GTP dependent and also previously correlated with adaptive response to stress [53]. The lower abundance of this protein possibly was dominated by high abundance of a suppressor CodY protein, since both require GTP for its activity.

When comparing the stationary phase of C and CL to their respective exponential phases (StaCL/ExpCL and StaC/ExpC), proteins such as 5-methylthioadenosine *S*-adenosylhomocysteine (SAM) nucleosidase (pfs CA_C2117) (salvage pathway) and M18 family aminopeptidase (apeA CA_C1091 and apeB CA_C0607) that recycle amino acids from peptides (preferably aspartate glutamate) were up-regulated during the stationary phase of both conditions. SAM nucleosidase (that produces universal quorum sensing autoinducer-2 [54]) was up-regulated during solventogenesis and may be the vital indicator of transition of phases [18].

This strongly suggests that metabolic activities are highly regulated at the transcription and translational level, not only in lignin conditions, but also during the transition from exponential to stationary phase.

### Protein expression changes associated with chemotaxis/cell division/sporulation/energy metabolism

Chemotaxis and motility are the vital functions in the lifestyle of many unicellular organisms and are metabolically costly processes. In this study, flagellin (flaC) and chemotaxis proteins (CheW and CheA) (CA_C2224 and CA_C2220) that trigger cellular motility in response to environmental conditions were significantly down-regulated in CL (ExpCL/ExpC and StaCL/StaC). These results are consistent with the literature which demonstrated loss of motility associated with no solvent production in *C. acetobutylicum* [55–57]. Interestingly, ESAT-6 antigen-like protein (CA_C0040), a vital early expression protein of secretion system VII was found in significantly high abundance in CL conditions. This indicates a strong and early response to lignin, which could have vital role in chemotaxis activity via flagellin in *C. acetobutylicum*. We speculate that there must be relation between ESAT-6, Chew/ChewA and flagellin that triggers the initial stress response with subsequent changes in transcription/translation sporulation/cell division proteins.

In this study, we identified seven differentially regulated proteins from the divisome (division complex). As *C. acetobutylicum* is closely related to *B. subtilis*, it can be speculated that the structural complex could be similar to the divisome in *B. subtilis*. Our data show most of these proteins were up-regulated during growth on CL compared to C, indicating significant changes in divisomes (ExpCL/ExpC) induced by lignin. In particular, protein DivIVA (CA_C2118), a vital self-recruiting protein and involved in chromosome segregation during sporulation [58], SepF (CA_C2120), Z ring forming protein and cell division protein FtsX (CA_C0498) and FtsZ (CA_C1693) were found to be at increased abundance. In previous studies, high abundance of these proteins was correlated to abnormal morphology [59] and delay in sporulation [58]. This is confirmed by the controller of the sporulation protein (Spo0A) being down-regulated in CL conditions (ExpCL/ExpC and StaCL/StaC). Sporulation Spo0A (CA_C2071) induces the ftsZ protein to allow cells to divide at transcriptional level and governs sporulation/solventogenesis at the transcriptional level [60, 61].

Evidence suggests that cell division and sporulation are concomitantly regulated by a stress response protein complex (ClpP, ClpX and ClpC and Lon protease) that works as a quality control and regulatory proteolysis pathway under stress conditions [62] to remove/stabilize defective or aggregated proteins. In this study, we found that several ATP-dependent proteins, ClpB (CA_C0959), ClpC (CA_C094, CA_C3189), DnaK (CA_C1282), Lon (CA_C2637), TerE (CA_C1412) and hsp18 (CA_C3714), were significantly down-regulated in lignin stress conditions. Therefore, we hypothesize that the quality control system of this bacterium fails to keep cells up to date, resulting in abnormal cell division/sporulation.

It has been demonstrated that the relative concentrations of nucleotides (particularly ATP and GTP) play important roles in cell physiology and regulation [63]. In *C. acetobutylicum*, glycolysis and acids production during the exponential phase are energy generation (ATP generation) steps [64]. In this work, seven subunits from ATP synthase were identified, including alpha, beta, gamma, epsilon and delta subunits. However, the relative abundance of these proteins was not significantly regulated in both conditions. This suggests that intracellular protons are mostly used for hydrogen production, thus limiting expenditure of ATP at substrate level phosphorylation [65]. Thus, it can be hypothesized that down-regulation of glycolysis and acid production decreased ATP synthesis in CL conditions to cumulatively affect cell regulation. WrbA family protein (multimeric flavodoxin) (CA_C3314) was found at high abundance in CL treatment (StaCL/StaC and StaCL/ExpCL) which is involved in electron transfer systems and only expresses during adaptive cell response to stress conditions [66].

It has been proposed that *C. acetobutylicum* possesses another energy conserving module based on NADH:ferredoxin oxidoreductase (rnf) and butyryl-CoA dehydrogenase complex (Bcd/etfAB), [67] also proposed in *Clostridium kluyveri* [68]. Interestingly, we identified butyryl-CoA dehydrogenase (Bcd) (CA_C2711), electron transfer flavoprotein (subunit etfA (CA_C2709) and etfB (CA_C2710) and probable NADH/NADPH oxidoreductase (CA_C1958) in both conditions, suggesting the presence of extra energy conserving modules in this bacterium.

## Conclusions

In conclusion, our study presents the most comprehensive analysis of the effect of lignin on cellular metabolism of *C. acetobutylicum*. This is the first time that the inhibitory effect of lignin on growth, morphology, ABE and H_2_ production and cellular functions was investigated and integrated. Glycolysis, fermentation and associated pathways were significantly repressed when lignin was present. Several proteins involved in the glycolysis and fermentation pathways were down-regulated in the presence of lignin concomitantly with lower ATP production. Lignin also suppressed the ATP-dependent Clp protease complex (which controls normal cell division) synthesis and activity resulting in a delay in sporulation and solventogenesis. Lignin imposed morphological adaptation since cellular stress associated with decreased ATP-dependent housekeeping activity and the cellular divisome were affected. Our main aim was to analyse the ‘lignin bottleneck’ by monitoring fermentation end products and associated changes in the proteome of *C. acetobutylicum* in response to lignin with a view on providing insights into lignocellulose as a feedstock for biofuel generation. Our results shed light on the breadth of the metabolic routes involved in the lignin response in a commercially valuable bacterium for future implementation for lignocellulosic biofuel generation.

## Methods

### Bacterial strain and growth conditions

All chemicals and reagents were purchased from Sigma-Aldrich (Poole, UK) unless otherwise specified. *C. acetobutylicum* ATCC 824 was procured from the German Collection of Microorganisms and Cell Cultures (DSMZ, Braunschweig, Germany) and was maintained anaerobically on medium as previously described by Lopez-Contreras et al. [69]. Briefly, the growth media contained 0.75 g L^−1^ KH_2_PO_4_, 0.75 g L^−1^ K_2_HPO_4_, 0.348 g L^−1^ MgSO_4_, 0.01 g L^−1^ MnSO_4_·H_2_O, 0.01 g L^−1^ FeSO_4_·7H_2_O, 1 g L^−1^ NaCl, 1.0 g L^−1^ cysteine chloride, 5 g L^−1^ yeast extract and 2 g L^−1^ (NH_4_)_2_SO_4_.

The cellobiose-only growth medium was prepared with 5 g L^−1^ cellobiose (hereafter denoted as C) whereas the cellobiose/lignin medium was prepared with 5 g L^−1^ cellobiose plus 1 g L^−1^ Kraft lignin (alkali, carboxylated lignin; Sigma-Aldrich; Cat. No. 470996-100G) (hereafter denoted as CL). Media were anaerobically prepared in the presence of 100 % nitrogen gas in 125-mL serum bottles and autoclaved. The culture media were seeded with 1 mL of 18-h-long cultures in which optical density (OD) at 600 nm was equal to 1.3. Cultures were incubated at 37 °C and growth curves were monitored at OD_600nm_ using an UltraSpec 2100 (Amersham Bioscience, GE Healthcare, Buckinghamshire, UK). The cellobiose concentration was estimated by the Anthrone method [70].

### Microscopic observations

Cell morphology was observed using Olympus microscope BX51 (Tokyo, Japan) fitted with a CapturePro 2.6-JENOPTIK Laser, Optik, System (GmbH, Germany) camera and an FEI Tecnai transmission electron microscope at an accelerating voltage of 80 kV. Electron micrographs were taken using a Gatan digital camera (Gatan, Oxon, UK).

### Fermentation end products analysis

Targeted fermentation products were identified and quantified as previously reported by Pham et al. [71]. Briefly, ethanol, butanol, acetic acid and butyric acid were detected and quantified using an Agilent 7890A gas chromatograph (GC) (Cheshire, UK) instrument equipped with a flame ionization detector (FID) and Stabbilwax (30 m × 0.25 mm ID × 0.25 µm df) fused silica column (Thames Restek, Buckinghamshire, UK). Aliquots (50 μL) were collected from four biological replicates, centrifuged at 17,000×*g* for 2 min and transferred to a GC vial, and then 2 μL of supernatant was injected into the column. The GC was controlled and automated by ChemStation (Agilent, Rev: 32.3.8) software. Each GC run was performed for 14 min using a temperature gradient (with a hold at 45 °C for 3 min, followed by a ramp at a rate of 15 °C/min to 120 °C, then 30 °C/min to 210 °C and finally a hold 1 min at 210 °C). Helium was used as the carrier gas at a flow rate of 20 mL min^−1^. The injector, detector and oven temperatures were 250, 350 and 120 °C, respectively. The concentrations of ethanol, butanol, acetic acid and butyric acid were estimated by comparing its retention time and peak area against standard curves of respective metabolite.

### Hydrogen gas estimation

Gas samples from four biological replicates were collected from the headspace culture bottles using 10 mL gas tight syringes at different interval times. At each time point, the sample was injected into a gas chromatograph TRACE 1300 (Thermo Scientific, Paisley, UK) equipped with a thermal conductivity detector (TCD) and a 250 μL of sample was injected into the column. Separation was achieved using a precolumn Haysep Q (60–80) column with 2 m × 1/16 SS packing connected with Molsieve 5A (60–80) column with 2 m × 1/16 SS packing. Argon was used as carrier at 36.25 psi pressure. Each GC run was performed for 13 min using a temperature gradient (with a hold at 50 °C for 2.5 min, followed by a ramp at a rate of 20 °C/min to 70 °C for 45 s, then hold at 70 °C for next 8.35 min and finally ramp of 1 min up to 150 °C). The detector and valve oven temperatures were 150 and 80 °C, respectively. The GC was controlled and automated by the Chromeleon software (Dionex, Version 7). The instrument was calibrated using hydrogen gas standards of 10, 30 and 40 % (*v/v*) from BOC (Guildford, Surrey, UK).

### Cell harvesting and protein extraction

Cells grown in C and CL media were harvested at mid-exponential (16 h) and late stationary (48 h) phases and pelleted by centrifugation at 10,000×*g* for 5 min at 4 °C. Cell pellets were washed twice with phosphate buffer saline (PBS) and once with 0.5 M triethylammonium bicarbonate (TEAB), pH 8.5 buffer. The cells were re-suspended in 600 μL lysis buffer [0.5 M TEAB containing 0.095 % (*v/v*) sodium dodecyl sulphate (SDS) and 5 μL protease inhibitor cocktail set II, pH 8.5] and 300 mg sterilized acid-washed glass beads (425–600 µm) were also added. Cell lysis was performed using a cell disruptor (Genie, VWR, UK) with 20 cycles of alternative 1 min vortexing and 1 min incubation on ice. Unbroken cells and cell debris were pelleted by centrifugation at 21,000×*g* for 90 min at 4 °C and the supernatant was transferred to a clean Eppendorf tube. Five microlitre of benzonase^®^ nuclease (1:100) was added to the supernatant to clarify the sample by degradation of nucleic acids. Proteins were acetone precipitated (approximately 16 h) at −20 °C (ratio sample: acetone = 1:4 *v/v*). Precipitated proteins were recovered by centrifugation at 17,000×*g* for 20 min at −9 °C and air dried. Finally, the pellet was dissolved in 0.5 M TEAB, pH 8.5 buffer containing 0.1 % (*v/v*) RapiGest SF (Waters, Milford, MA, USA). The total protein concentration was estimated by the Bradford assay according to the manufacturer’s protocol.

### Quantitative workflow and iTRAQ labelling

iTRAQ 8-plex labelling was performed for samples according to the manufacturer’s protocol (8-plex iTRAQ reagent Multiplex kit, ABSciex, USA). Briefly, 100 µg of proteins from each sample was first reduced with 1 µL of 50 mM Tris (2-carboxyethyl) phosphine hydrochloride and incubated at 60 °C for 1 h. Samples were then alkylated using 1 μL of 200 mM methyl methanethiosulfonate at room temperature for 10 min. Subsequently, proteins were digested with trypsin (Promega, UK) at a ratio of 1:50 (trypsin:protein) overnight at 37 °C. An independent biological duplicate was used for each phenotype. Each biological phenotype was labelled with relevant iTRAQ reagents as shown in Fig. 1. Labelled peptides were acidified with trifluoroacetic acid to precipitate RapiGest SF and removed by centrifugation at 17,000×*g* for 5 min at 4 °C. iTRAQ-labelled peptides, present in the eight sample supernatants, were combined and concentrated by a vacuum concentration (Scanvac, Lynge, Denmark).

### Off-line fractionation

iTRAQ-labelled peptides were off-line fractionated using hydrophilic interaction liquid chromatography (HILIC) on an Agilent 1100 HPLC (Berkshire, UK). Dried peptides were re-suspended in HILIC buffer A [80 % (*v/v*) acetonitrile (ACN), 10 mM ammonium formate, pH 3] and loaded to polyhydroxyethyl-A column, 5 μm pore size, 100 mm length, 4.6 mm ID (Poly LC, MD, USA) at a flow rate of 0.5 mL/min using an UVD170U detector (Dionex/LC packings, The Netherlands) at 280 nm. HILIC buffer A and buffer B [5 % (*v/v*) ACN, 10 mM ammonium formate, pH 5] were used to perform a 90 min gradient of 0 % B for 10 min, 0–20 % B for 15 min, 20–40 % for 30 min, 40–60 % for 15 min, 60–100 % B for 5 min, and 100 % A for 15 min. Fractionation and chromatogram were monitored through Chromeleon software (Thermo, Hemel Hempstead, UK). Fifty-eight fractions were collected at one-minute intervals and dried by vacuum concentration (Scanvac, Lynge, Denmark) before tandem mass spectrometric analysis.

### Mass spectrometry analysis

HILIC fractions were solubilised in reverse phase (RP) buffer A [3 % (*v/v*) acetonitrile, 0.1 %(*v/v*) formic acid (FA)] before submitting to a QStarXL Hybrid ESI Quadrupole Time-of-Flight Tandem mass spectrometer [Applied Biosystems (now ABSciex), Famingham, MA] coupled with an online Ultimate 3000 HPLC system (Dionex, Surrey UK). Reverse-phase peptide separation was performed on a C18 Acclaim^®^ PepMap100 column (3 μm, 100 Å, 15 cm) at a flow rate of 300 nL min^−1^. A 120-min linear gradient was applied; RP buffer A and RP buffer B [97 % (*v/v*) ACN, 0.1 % (*v/v*) FA] were used as follows: 0–3 % B for 5 min, 3–35 % B for 90 min, 35–90 % of B for 0.5 min, 90 % of B for 6.5 min, finally 3 % of buffer B for 18 min. Data were acquired in positive ion mode in the data-dependent acquisition mode. The MS survey scan was set to cover the *m/z* range of 350–1800 Th and the MS/MS survey scan was set to the *m/z* range of 100–1600 Th using Analyst^®^ QS 2.0 software (ABSciex, Famingham, MA). Peptides of charge +2, +3, +4 (intensity binning) for each TOF–MS scan (400–1250 *m/z*) were dynamically selected and isolated for MS/MS fragment ion scans (100–1600 *m/z*). Two RP-HPLC–MS runs per HILIC fraction were performed.

### Data interpretation and protein identification

The generated tandem MS data files (.wiff) from the QSTAR XL were converted into mascot generic files (.mgf) via the mascot.dll embedded script (V1.6) coupled with Analyst QS v. 1.1.1 (ABSciex, MDS-Sciex). Peptide identification was performed using an in-house Phenyx algorithm cluster (Binary version 2.6, Genebio Geneva, Switzerland) using the *C. acetobutylicum* ATCC 824 database (taxon ID: 272562) containing 3825 protein sequences, which was downloaded from Uniprot (http://www.uniprot.org, June 2014). Searches were also conducted against a forward/reverse concatenated database to determine the false discovery rate (FDR). Search parameters considered trypsin as enzyme allowing a maximum of two missed cleavage sites. As fixed modifications, 8-plex iTRAQ of lysine (K) and peptide N-terminus (+304 Th) and methylthio of cysteine residues (+46 Th) were included in the search. Oxidation of methionine (+16 Th) and deamidation (N and Q) (−1 Th) were also considered as variable modifications. Mass tolerances for peptide identification were set to 0.5 and 0.2 Da for MS and MS/MS, respectively. Thresholds for identified peptides were set to a minimal *Z*-score of 5.0, a *p* value of 10^−4^, an AC score of 5 and peptide length of minimum six amino acids. Phenyx results were exported to Excel *.xls format and 1 % FDR with two unique peptides per protein was used as the confident protein identifier. iTRAQ ratios used for relative quantification were determined by applying an in-house data analysis pipeline [72]. Isotopic and median corrections were applied to the reporter ion’s intensities to compensate systematic errors between labels. Ultimately, a statistical method, described by Pham et al. [73] was applied to determine which proteins were significantly regulated at *p* value <0.05. Cluster analysis and Principal Component Analysis were performed using Mathematica 10.2 software (Wolfram Research, Oxfordshire, UK).

## Additional files

10.1186/s13068-016-0523-0 Metabolites data. The detailed metabolites information included cellobiose consumption, cell dry biomass, acetic acid, butyric acid, ethanol, butanol and hydrogen gas are provided in separate excel file “Supporting information metabolites concentrations”.
10.1186/s13068-016-0523-0 Proteomic data. The detailed proteomics information included, Phenyx ID, quantification ID and fold changes (iTRAQ ratio; ExpCL vs ExpC, StaCL/StaC, StaC vs ExpC and StaCL vs ExpC) are provided in separate excel file “Supporting information proteomics iTRAQ”.
10.1186/s13068-016-0523-0 Alterations in relative abundance of protein expressions (iTRAQ ratio representing fold changes) in other metabolic pathways during growth on C and Cl. **Table S1.** Comparing protein regulations between C (control) and CL at exponential (Exp) and stationary (Sta) phases. Table includes identified proteins, fold change and *p* value for each comparison.

## Abbreviations

ABE: acetone-butanol-ethanol
iTRAQ: isobaric tags for relative and absolute quantitation
FDR: false discovery rate
C: cellobiose
CL: cellobiose plus lignin
H_2_: molecular hydrogen
PCA: principal component analysis
PEP: phosphoenolpyruvate
PTS: phosphotransferase system
KDPG: 2-keto-3-deoxy-6-phosphogluconate
EMP: Embden–Meyerhof–Parnas
ED: Entner–Doudoroff
GC: gas chromatography
TCA: tricarboxylic acid
HydA: hydrogenase
SAM: *S*-adenosylmethionine
OD: optical density
FID: flame ionization detector
TCD: thermal conductivity detector
PBS: phosphate buffer saline
TEAB: triethylammonium bicarbonate
SDS: sodium dodecyl sulphate
FA: formic acid
HILIC: hydrophilic interaction liquid chromatography
ACN: acetonitrile
HPLC: high-performance liquid chromatography
MS: mass spectrometry
RP: reverse phase
