# Supplementary material for: Quantitative proteomic analysis of the influence of lignin on biofuel production by Clostridium acetobutylicum ATCC 824
Source: Biotechnol Biofuels. 2016 May 31;9:113. doi: 10.1186/s13068-016-0523-0 (PMC4886415; doi:10.1186/s13068-016-0523-0)
Supplement: Supplementary file 1 — 10.1186/s13068-016-0523-0 Metabolites data. The detailed metabolites information included cellobiose consumption, cell dry biomass, acetic acid, butyric acid, ethanol, butanol and hydrogen gas are provided in separate excel file “Supporting information metabolites concentrations”. [file 13068_2016_523_MOESM1_ESM.docx]

Supplementary materials

Quantitative proteomics reveals metabolic responses to lignin by *Clostridium acetobutylicum* ATCC 824

Mahendra P. Raut^1^, Narciso Couto^1^, Trong K. Pham^1^, Caroline Evans^1^, Josselin Noirel^1, 2^, Phillip C. Wright^1,^*

^1^ The ChELSI Institute, Department of Chemical and Biological Engineering, University of Sheffield, Mappin Street, Sheffield, S1 3JD, UK

^2^ Chaire de bioinformatique, LGBA, Conservatoire national des arts et métiers, 75003 Paris, France

*Corresponding author: Phillip C. Wright. Tel: +44 114 222 7577, Fax: +44 114 222 7501, E-mail: p.c.wright@sheffield.ac.uk

E. mail: Mahendra P. Raut – m.raut@sheffield.ac.uk; Narciso Couto - n.couto@sheffield.ac.uk; Trong K. Pham - khoa.pham@sheffield.ac.uk; Caroline Evans - caroline.evans@sheffield.ac.uk; Josselin Noirel - j.noirel@sheffield.ac.uk; Phillip C. Wright* - p.c.wright@sheffield.ac.uk


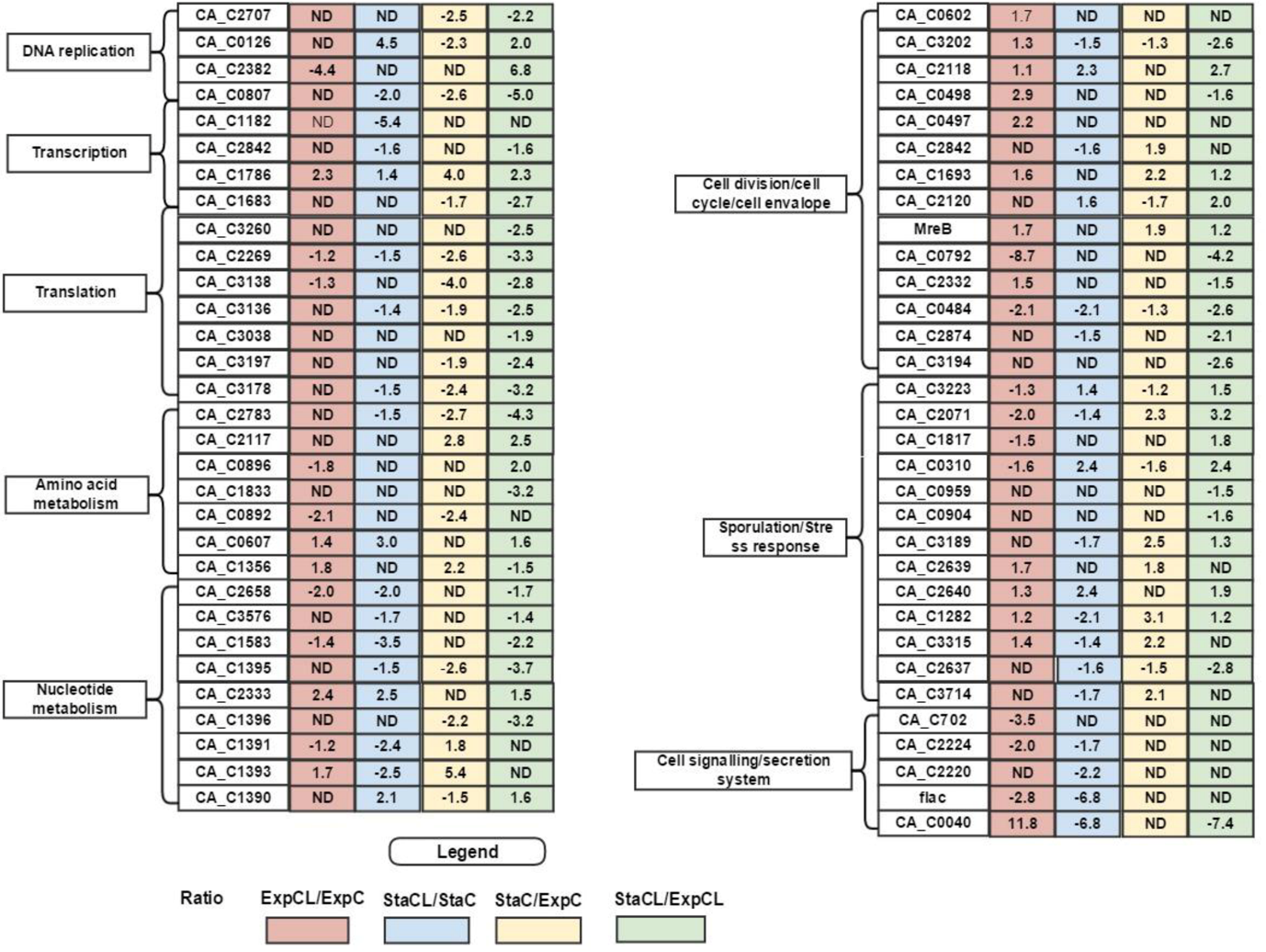


**Fig. S1 –** iTRAQ fold changes in protein expression levels during growth on C and CL. (Comparing ratio; ExpCL/ExpC (Red), StaCL/StaC (Blue), StaC/ExpC (Yellow) and StaCL/ExpC (Green)). **DNA metabolism**: CAC2707, 8-oxoguanine-glycoxylase; CA_C0126 nucleotide associated protein; CA_C2382, single standed DNA binding protein (ssb); CA_C0807, cold shock protein; **Transcription**: CA_C1182, phage related protein; CA_C2842, transcription assessor protein (TEX); CA_C1786 GTP sensing transcriptional pleiotropic repressor (CodY); CA_1683 rinuclease J (rnj); **Translation**: CA_C3260, Aspargine-tRNA ligase (asnS); CA_C2269, Aspartate-tRNA ligase (aspS); CA_C3138, Elongation factor G (FusA); CA_C3136, Elongation factor Tu; CA_C3038 isoleusine-tRNA ligase (IleS); CA_C3197, Lysine-tRNA ligase (LysS); CA_C3178, Proline-tRNA ligase (ProS); **Amino acid metabolism**: CA_C2783, O-acetylhomoserine sulfhydrylase (cysD); CA_C2117, 5'-methylthioadenosine/S-adenosylhomocysteine nucleosidase (pfs); CA_C0896, Chorismate synthase (aroC), CAC1833, Cystathionine β lyase family; CA_C0892, DHAP synthase; CA_C0607), M18 family aminopeptidase (apeB); CA_C1356, Thiamine synthase enzyme (ThiH); **Nucleotide metabolism:** CA_C2658, Glutamine synthase III (glnA); CA_C3576, 2-nitropropane dioxygenase; CA_C1583, P-loop ATPase; CA_C1396, Bifunctional purine biosynthesis (purH); CA_C1396, Phosphoribosylamine glycine ligase (purD); CA_C1391, SAICAR synthetase (purC); CA_C1393, AIR synthase (purM); CA_C1390, N5-CAIR mutase (purE); **Cell division/cell envelope**: CA_C0602 and CA_C3202, ATP dependant zinc metalloprotease (FtsH); CA_C2118, Cell division ATP binding; CA_C0498,CA_C497, CA_C1693, cell division protein (FtsX, FtsE, FtsZ ); CA_C2120, Site determining protein (sepF); CA_C1242, cell morphology (mreB); CA_C0792, aminotransferase; CA_C2332, dTDP-glucose 4,6-dehydratase (spsJ), CA_C0484, Phosphoglucosamine mutase (glmM); CA_C2874, UDP-GlcNAc-2-epimerase; CA_C3194, CA_C3194, D-glutamic acid-adding enzyme (MurD): **Sporulation/Stress response**: CA_C3223, Septation protein (SpoVG); CA_C2071, Stage 0 sporulation protein stage A (Spo0A); CA_C1817, Stage V sporulation protein (spoVS); CA_C0310, sporulation/stationary gene regulatory protein (abrB); CA_C0959,CA_C0904,CA_C3189, CA_C2639, CA_C2640 Chaperone protein (ClpB, ClpC ClpX ClpP); CA_1282, CA_C3315, Chaperone protein (DnaK htpG; CA_C2637, Lon protein (lon); CA_C3714, 18 kdA heat shock protein; **Cell signalling/ secretion system**: CA_C0702, Med/BMP family; CA_C2224, Chemotaxis protein (CheW); CA_C2220, Histidine kinase (CheA); CA_C0040, Flagellin (flac); ESAT-6 like protein. (Fold change in protein expression: negative values indicates low abundance of proteins and positive values indicates high abundance of proteins)

**Table S1**  List of differentially regulated proteins between C (control) and CL at exponential (Exp) and stationary (Sta) phase. Table summarising identified proteins, fold change, p-value for each comparison. A negative value indicates down-regulation and positive values indicates up-regulation of proteins. ExpCL; Exponential phase of cellobiose plus lignin grown cells, StaCL; stationary phase of cellobiose plus lignin grown cells, ExpC; exponential phase of cellobiose only grown cells and StaCL; stationary phase of cellobiose only grown cells. ND; Not determined.

| **Classification** | **Protein Name** | **Gene Locus** | **Fold changes/Ratio** | | | |
| --- | --- | --- | --- | --- | --- | --- |
|  |  |  | **ExpCL/ExpC** | **StaCL/**  **StaC** | **StacC/ExpC** | **StaCL/ExpC** |
| **Energy Metabolism** | | | | | | |
| Q9Z686 | ATP synthase epsilon chain | atpC CA_C2864 | 1.2 | ND | 1.8 | ND |
| Q9Z688 | ATP synthase gamma | atpG CA_C2866 | ND | ND | -2.1 | -2.1 |
| Q9Z689 | ATP synthase subunit alpha | atpA CA_C2867 | ND | -1.5 | -1.5 | -1.9 |
| O05098 | ATP synthase subunit b | atpF atpB CA_C2869 | 1.5 | ND | ND | -1.3 |
| Q9Z687 | ATP synthase subunit beta | atpD CA_C2865 | 1.2 | -1.3 | 1.8 | 1.1 |
| Q9Z687 | ATP synthase subunit beta | atpD CA_C2865 | -1.3 | -1.3 | 1.8 | 1.1 |
| Q9Z690 | ATP synthase subunit | atpH CA_C2868 | -1.7 | ND | ND | -1.7 |
| Q97KD9 | Pyruvate-formate lyase | pflB CA_C0980 | ND | ND | -2.2 | -2.3 |
| Q97EM7 | Thioredoxin | CA_C3083 | -2.0 | -1.5 | -2.7 | -2.0 |
| Q97KH5 | Transketolase | tkt CA_C0944 | -1.4 | ND | -1.2 | -1.7 |
| Q97J57 | UDP-glucose 4-epimerase | galE CA_C1429 | -1.7 | ND | -4.2 | -1.5 |
| Q97MU7 | Ferredoxin-nitrite reductase | CA_C0094 | -1.3 | ND | ND | -1.6 |
| **Energy conservation** | | | | | | |
| Q97K92 | Flavo-diiron protein FprA1 | fprA1 CA_C1027 | -1.7 | -2.0 | 2.6 | 2.2 |
| Q97DX9 | Multimeric flavodoxin WrbA family | CA_C3341 | -1.1 | 2.4 | ND | 1.5 |
| P52039 | Electron transfer flavoprotein subunit alpha (Alpha-ETF) | etfA CA_C2709 | ND | -1.8 | 2.0 | ND |
| P52040 | Electron transfer flavoprotein subunit beta (Beta-ETF) | etfB CA_C2710 | ND | -1.8 | 2.5 | 1.4 |
| **DNA replication** | | | | | | |
| Q97FM4 | 8-oxoguanine-DNA-glycosylase | CA_C2707 | ND | ND | -2.5 | -2.2 |
| Q97EA3 | DNA binding protein HU | hbs CA_C3211 | ND | ND | ND | 1.2 |
| P94605 | DNA gyrase subunit A | gyrA CA_C0007 | ND | -1.6 | 1.8 | ND |
| Q97N34 | DNA polymerase III subunit beta | dnaN CA_C0002 | ND | 1.7 | -1.8 | ND |
| Q97MG8 | Inactivated predicted Zn-dependent protease, PMBA ortholog | CA_C0230 | -1.2 | ND | -1.7 | ND |
| Q97MR5 | Nucleoid-associated protein | CA_C0126 | ND | 4.5 | -2.3 | 2.0 |
| Q97CW1 | Predicted RNA-binding protein Jag, SpoIIIJ-associated | jag CA_C3735 | ND | ND | 1.9 | 1.6 |
| Q97MG9 | Predicted Zn-dependent proteases, TLDD ortholog | CA_C0229 | ND | ND | ND | -1.5 |
| Q97H75 | Probable manganese-dependent inorganic pyrophosphatase | ppaC CA_C2138 | -1.1 | -1.3 | ND | -1.1 |
| Q97GI5 | Single-strand DNA-binding protein, ssb | ssb CA_C2382 | -4.4 | ND | ND | 6.8 |
| Q97CW7 | Stage 0 sporulation J, ParB family of DNA-binding proteins | spoOJ CA_C3729 | ND | ND | 1.9 | 1.8 |
| **Transcription** | | | | | | |
| Q97EW6 | Cold shock protein | csp CA_C2990 | -1.7 | ND | -1.8 | -1.8 |
| Q97KV9 | Cold shock protein | CA_C0807 | ND | -2.0 | -2.6 | -5.0 |
| Q97EK6 | DNA-directed RNA polymerase subunit alpha | rpoA CA_C3104 | 1.1 | -1.1 | 1.3 | ND |
| Q97EG9 | DNA-directed RNA polymerase subunit beta ( | rpoB CA_C3143 | -1.1 | -1.5 | -3.1 | -4.3 |
| Q97EH0 | DNA-directed RNA polymerase subunit beta' | rpoC CA_C3142 | ND | ND | -3.3 | -3.6 |
| Q97IC9 | DNA-directed RNA polymerase subunit omega | rpoZ CA_C1719 | ND | ND | ND | 1.3 |
| Q97JU1 | Phage related protein, YorG B.subtilis homolog | CA_C1182 | ND | -5.4 | ND | ND |
| Q97M85 | Phage shock protein A | CA_C0313 | 1.5 | 1.4 | 1.8 | 1.7 |
| Q97I45 | Polyribonucleotide nucleotidyltransferase | pnp CA_C1808 | ND | ND | -2.9 | -2.9 |
| Q97GS1 | Probable transcriptional regulatory protein | CA_C2295 | ND | ND | 2.3 | 1.5 |
| Q97IF8 | Ribonuclease J | rnj CA_C1683 | ND | ND | -1.7 | -2.7 |
| P33656 | RNA polymerase sigma factor SigA | sigA rpoD CA_C1300 | 1.2 | ND | 1.5 | ND |
| Q97F98 | Transcription accessory protein TEX, | CA_C2842 | ND | -1.6 | ND | -1.6 |
| Q97I54 | Transcription termination/antitermination protein NusA | nusA CA_C1799 | ND | ND | -1.5 | -1.9 |
| Q97EG4 | Transcription termination/antitermination protein NusG | nusG CA_C3149 | 1.9 | ND | 1.9 | 2.0 |
| Q97KE2 | Transcriptional regulator, Lrp family | asnC CA_C0977 | 1.5 | 3.4 | ND | 1.6 |
| Q97E33 | Transcriptional regulator, MarR/EmrR family | CA_C3283 | 1.3 | ND | ND | ND |
| Q97I67 | GTP-sensing transcriptional pleiotropic repressor CodY | codY CA_C1786 | 2.3 | 1.4 | 4.0 | 2.3 |
| **Translation** | | | | | | |
| Q97E56 | Asparagine--tRNA ligase | asnS CA_C3260 | ND | ND | ND | -2.5 |
| Q97GU6 | Aspartate--tRNA ligase | aspS CA_C2269 | -1.2 | -1.5 | -2.6 | -3.3 |
| Q97FQ8 | Aspartyl/glutamyl-tRNA(Asn/Gln) amidotransferase subunit B 1 | gatB1 CA_C2669 | ND | -1.4 | 1.5 | ND |
| Q97EH4 | Elongation factor G (EF-G) | fusA CA_C3138 | -1.3 | ND | -4.0 | -2.8 |
| Q97HB8 | Elongation factor P (EF-P) | efp CA_C2094 | ND | ND | ND | 1.2 |
| Q97I65 | Elongation factor Ts (EF-Ts) | tsf CA_C1788 | ND | -1.3 | ND | -1.1 |
| Q97EH5 | Elongation factor Tu (EF-Tu) | tuf CA_C3136 | ND | -1.4 | -1.9 | -2.5 |
| Q97KC9 | Glutamate--tRNA ligase | gltX CA_C0990 | -1.3 | ND | ND | ND |
| Q97ES0 | Isoleucine--tRNA ligase | ileS CA_C3038 | ND | ND | ND | -1.9 |
| Q97EB7 | Lysine--tRNA ligase | lysS CA_C3197 | 1.5 | ND | ND | -1.1 |
| Q97EW5 | Methionine--tRNA ligase | metG CA_C2991 | ND | ND | -1.9 | -2.4 |
| Q97G09 | NifU-related domain containing protein | CA_C2565 | -1.2 | -1.2 | -1.6 | -1.9 |
| Q97ED5 | Proline--tRNA ligase | proS CA_C3178 | ND | -1.5 | -2.4 | -3.2 |
| Q97H79 | Ribosome-binding ATPase YchF | ychF CA_C2134 | ND | ND | -1.7 | ND |
| Q97IF7 | TYPA/BIPA type GTPase | CA_C1684 | -1.3 | -1.4 | -2.3 | -2.5 |
| Q97GG8 | Valine--tRNA ligase | valS CA_C2399 | ND | ND | -1.8 | -2.0 |
| **Ribosomal proteins** | | | | | | |
| Q97EH7 | 30S ribosomal protein S10 | rpsJ CA_C3134 | ND | ND | 1.4 | 1.7 |
| Q97EK4 | 30S ribosomal protein S11 | rpsK CA_C3106 | 1.5 | ND | 2.3 | 1.4 |
| Q97I46 | 30S ribosomal protein S15 | rpsO CA_C1807 | ND | 1.3 | 1.3 | 1.4 |
| Q97CX4 | 30S ribosomal protein S18 | rpsR CA_C3722 | ND | 2.2 | ND | 1.6 |
| Q97I66 | 30S ribosomal protein S2 | rpsB CA_C1787 | 1.3 | -1.3 | 1.7 | ND |
| Q97JK0 | 30S ribosomal protein S20 | rpsT CA_C1274 | 1.3 | ND | ND | ND |
| Q97EK5 | 30S ribosomal protein S4 A | rspD1 CA_C3105 | ND | -1.2 | ND | -1.3 |
| Q97EJ5 | 30S ribosomal protein S5 | rpsE CA_C3116 | 1.3 | ND | 1.3 | ND |
| Q97EH3 | 30S ribosomal protein S7 | rpsG CA_C3139 | 1.4 | ND | 1.9 | 1.2 |
| Q97EJ2 | 30S ribosomal protein S8 | rpsH CA_C3119 | 1.7 | -1.3 | 2.8 | 1.4 |
| Q97EL3 | 30S ribosomal protein S9 | rpsI CA_C3097 | 2.5 | ND | 1.8 | ND |
| Q97EG6 | 50S ribosomal protein L1 | rplA CA_C3147 | 1.4 | -1.5 | 2.0 | ND |
| Q97EG7 | 50S ribosomal protein L10 | rplJ CA_C3146 | 1.2 | 1.1 | 1.3 | 1.3 |
| Q97EG5 | 50S ribosomal protein L11 | rplK CA_C3148 | 1.8 | 1.4 | 2.8 | 2.1 |
| Q97EL2 | 50S ribosomal protein L13 | rplM CA_C3098 | 1.2 | ND | 1.5 | 1.2 |
| Q97EI8 | 50S ribosomal protein L14 | rplN CA_C3123 | 1.3 | 1.3 | 1.6 | 1.7 |
| Q97EJ7 | 50S ribosomal protein L15 | rplO CA_C3114 | ND | ND | ND | 1.1 |
| Q97I93 | 50S ribosomal protein L19 | rplS CA_C1759 | 1.5 | ND | 2.2 | 1.2 |
| Q97EI1 | 50S ribosomal protein L2 | rplB CA_C3130 | 2.3 | ND | 2.5 | ND |
| Q97GK7 | 50S ribosomal protein L20 | rplT CA_C2359 | ND | -1.2 | 2.1 | 1.7 |
| Q97JL7 | 50S ribosomal protein L21 | rplU CA_C1257 | 1.4 | ND | ND | 1.6 |
| Q97EI3 | 50S ribosomal protein L22 | rplV CA_C3128 | 1.9 | ND | 1.7 | ND |
| Q97EI0 | 50S ribosomal protein L23 | rplW CA_C3131 | -1.2 | 1.3 | ND | 1.6 |
| Q97EI9 | 50S ribosomal protein L24 | rplX CA_C3122 | 1.5 | ND | ND | 1.4 |
| Q97IB7 | 50S ribosomal protein L28 | rpmB CA_C1733 | ND | ND | -3.5 | -1.9 |
| Q97EH8 | 50S ribosomal protein L3 | rplC CA_C3133 | 1.5 | ND | ND | ND |
| Q97EJ6 | 50S ribosomal protein L30 | rpmD CA_C3115 | -1.4 | ND | -2.3 | -1.3 |
| Q97F64 | 50S ribosomal protein L31 | rpmE CA_C2888 | -1.4 | ND | -2.3 | ND |
| Q97EH9 | 50S ribosomal protein L4 | rplD CA_C3132 | 1.7 | 1.9 | -1.4 | -1.2 |
| Q97EJ0 | 50S ribosomal protein L5 | rplE CA_C3121 | 1.4 | -1.1 | 2.2 | 1.4 |
| Q97EJ3 | 50S ribosomal protein L6 | rplF CA_C3118 | 1.1 | ND | 1.8 | 1.6 |
| Q97EG8 | 50S ribosomal protein L7/L12 | rplL CA_C3145 | ND | ND | 1.5 | 1.5 |
| Q97CX9 | 50S ribosomal protein L9 | rplI CA_C3717 | ND | 1.5 | 2.3 | 2.3 |
| **Transportation** | | | | | | |
| Q97MP5 | ABC transporter, ATP-binding protein | CA_C0147 | ND | ND | 1.9 | 1.5 |
| Q97E28 | Iron-regulated ABC transporter ATPase subunit | CA_C3288 | ND | -1.6 | 1.5 | -1.1 |
| Q97E27 | Iron-regulated ABC-type transporter membrane component (SufB) | CA_C3289 | ND | ND | 1.6 | 1.6 |
| Q97D48 | Oligopeptide ABC transporter, periplasmic substrate-binding component | oppA CA_C3632 | ND | -4.3 | 4.8 | ND |
| Q97EL6 | Uncharacterized consrved protein, associated with phosphate permease | CA_C3094 | 1.7 | ND | 2.3 | 1.7 |
| Q97K67 | Membrane protease subunit, stomatin/prohibitin homolog | CA_C1052 | 1.1 | 1.5 | -1.8 | -1.3 |
| Q97IF9 | Ferric uptake regulation protein | CA_C1682 | ND | 1.5 | ND | ND |
| **Amino acid metabolism** | | | | | | |
| Q97FF7 | O-acetylhomoserine sulfhydrylase | cysD CA_C2783 | ND | -1.5 | -2.7 | -4.3 |
| Q97H96 | 5'-methylthioadenosine/S-adenosylhomocysteine nucleosidase | pfs CA_C2117 | ND | ND | 2.8 | 2.5 |
| Q97I35 | Aspartate Aminotransferase | aspB CA_C1819 | -1.5 | ND | -6.3 | -5.3 |
| Q97LJ2 | Aspartate semialdehyde dehydrogenase (Gene asd) | asd CA_C0568 | ND | ND | -3.2 | -2.4 |
| Q97FT3 | Carbamoyl-phosphate synthase large chain | carB CA_C2644 | ND | -1.3 | ND | -2.0 |
| Q97KM1 | Chorismate synthase (CS) | aroC CA_C0896 | -1.8 | ND | ND | 2.0 |
| Q97LI3 | Cobalamine-dependent methionine synthase I | metH CA_C0578 | ND | -1.6 | ND | ND |
| Q97I22 | Cystathionine beta-lyase family protein, YNBB B.subtilis ortholog | CA_C1833 | ND | ND | ND | -3.2 |
| Q97GY0 | Cysteine synthase | cysK CA_C2235 | ND | ND | ND | 1.3 |
| Q97KM5 | DAHP synthase related protein | CA_C0892 | -2.1 | ND | -2.4 |  |
| Q97K30 | Probable M18 family aminopeptidase 1 | apeA CA_C1091 | ND | -1.2 | 2.7 | 1.6 |
| Q97LF4 | Probable M18 family aminopeptidase 2 | apeB CA_C0607 | 1.4 | 3.0 | ND | 1.6 |
| Q97E64 | Pyrroline-5-carboxylate reductase (P5C reductase) | proC CA_C3252 | -2.1 | -2.1 | ND | -1.8 |
| Q97JD0 | Thiamine biosynthesis enzyme ThiH | thiH CA_C1356 | 1.8 | ND | 2.2 | -1.5 |
| Q97J07 | Branched-chain-amino-acid transaminase (IlvE) | ilvE CA_C1479 | -1.2 | ND | -2.8 | -2.4 |
| Q97MV0 | Ketol-acid reductoisomerase | ilvC CA_C0091 | -1.3 | 1.9 | -3.3 | -1.3 |
| **Cell division/cell cycle/cell envalope** | | | | | | |
| Q97EB2 | ATP-dependent zinc metalloprotease FtsH | ftsH CA_C3202 | 1.3 | -1.5 | -1.3 | -2.6 |
| Q97LF9 | ATP-dependent zinc metalloprotease FtsH | ftsH CA_C0602 | 1.7 | ND | ND | ND |
| Q97LQ7 | Cell division ATP-binding protein | ftsE CA_C0497 | 2.2 | ND | 1.9 | ND |
| Q97H95 | Cell division protein DivIVA | CA_C2118 | 1.1 | 2.3 | ND | 2.7 |
| Q97LQ6 | Cell division protein FtsX | ftsX CA_C0498 | 2.9 | ND | ND | -1.6 |
| Q97IE9 | Cell division protein FtsZ | ftsZ CA_C1693 | 1.6 | ND | 2.2 | 1.2 |
| Q97H93 | Cell division protein SepF | sepF CA_C2120 | ND | 1.6 | ND | 2.0 |
| Q97JM4 | Site-determining protein | minD CA_C1249 | 1.4 | ND | 2.5 | 1.7 |
| Q97GN1 | UTP--glucose-1-phosphate uridylyltransferase | CA_C2335 | 1.7 | ND | ND | -1.5 |
| Q97GQ1 | DTDP-4-dehydrorhamnose reductase, rfbD ortholog | CA_C2315 | ND | ND | ND | -2.1 |
| Q97GN4 | dTDP-glucose 4,6-dehydratase | spsJ CA_C2332 | 1.5 | ND | ND | -1.6 |
| Q97LS0 | Phosphoglucosamine mutase | glmM CA_C0484 | -2.1 | -2.1 | -1.3 | -2.6 |
| P45360 | Putative UDP-N-acetylglucosamine 2-epimerase | CA_C2874 | ND | -1.5 | ND | -2.1 |
| Q97HC7 | Uncharacterized protein from alkaline shock protein family, YQHY B.subtilis ortholog | CA_C2085 | 1.3 | ND | 3.6 | 2.7 |
| Q97JN1 | MreB | mreB CA_C1242 | 1.7 | ND | 1.9 | 1.2 |
| Q97M76 | Sensory protein, containing EAL-domain | CA_C0322 | 1.4 | ND | 2.4 | 1.7 |
| Q97FT1 | Signal peptidase I | sipS CA_C2646 | -1.2 | ND | ND | 1.6 |
| Q97F58 | D-alanine--D-alanine ligase | ddl CA_C2895 | 1.3 | -1.2 | 1.6 | ND |
| Q97KX4 | D-amino acid aminotransferase | CA_C0792 | -8.7 | ND | ND | -4.2 |
| Q97DD9 | UDP-N-acetylglucosamine 1-carboxyvinyltransferase 2 | murA2 murZ CA_C3539 | ND | ND | -2.7 | -2.9 |
| Q97EB9 | UDP-N-acetylmuramoylalanine--D-glutamate ligase | murD CA_C3194 | ND | ND | ND | -2.6 |
| Q97H84 | UDP-N-acetylmuramoyl-L-alanyl-D-glutamate--2,6-diaminopimelate ligase 1 | murE1 CA_C2129 | ND | ND | -3.2 | -3.8 |
| O69136 | Flagellin | flaC | -2.8 | -6.8 | ND | ND |
| **Sporulation/Stress response** | | | | | | |
| Q97E91 | Putative septation protein SpoVG | spoVG CA_C3223 | -1.3 | 1.4 | -1.2 | 1.5 |
| P58253 | Stage 0 sporulation protein A homolog | spo0A CA_C2071 | -2.0 | -1.4 | 2.3 | 3.2 |
| Q97E52 | Uncharacterized conserved protein, YTFJ B.subtilis ortholog | CA_C3264 | ND | ND | 1.7 | 2.4 |
| Q97I37 | Stage V sporulation protein, spoVS | CA_C1817 | -1.5 | ND | ND | 1.8 |
| Q97M88 | Regulators of stationary/sporulation gene expression, abrB B.subtilis ortholog | abrB CA_C0310 | -1.6 | 2.4 | -1.6 | 2.4 |
| Q97KG0 | Chaperone protein ClpB | clpB CA_C0959 | ND | ND | ND | -1.5 |
| Q97KL3 | ATPase with chaperon activity, two ATP-binding domains, ClpC orthologs | CA_C0904 | ND | ND | ND | -1.6 |
| Q97EC4 | ATPases with chaperone activity clpC, two ATP-binding domain | clpC CA_C3189 | ND | -1.7 | 2.5 | 1.3 |
| Q97FT7 | ATP-dependent Clp protease ATP-binding subunit ClpX | clpX CA_C2639 | 1.7 | ND | 1.8 | ND |
| P58276 | ATP-dependent Clp protease proteolytic subunit | clpP CA_C2640 | 1.3 | 2.4 | ND | 1.9 |
| P30721 | Chaperone protein DnaK (HSP70) | dnaK CA_C1282 | 1.2 | -2.1 | 3.1 | 1.2 |
| Q97E05 | Chaperone protein HtpG | htpG CA_C3315 | 1.4 | -1.4 | 2.2 | ND |
| Q97FT9 | Lon protease | lon CA_C2637 | ND | -1.6 | -1.5 | -2.8 |
| Q97J74 | Methyl methane sulfonate/mytomycin C/UV resistance protein, GSP18 (YCEE) B.subtilis ortholog, TerE family protein | cdrC CA_C1412 | 2.3 | ND | ND | -2.6 |
| Q97IB6 | Uncharacterized, alkaline shock induced protein | CA_C1734 | ND | ND | 2.7 | 2.4 |
| Q03928 | 18 kDa heat shock protein (HSP 18) | hsp18 CA_C3714 | ND | -1.7 | 2.1 | ND |
| **cell signalling/secretion system** | |  |  |  |  |  |
| Q97FF2 | Xaa-Pro aminopeptidase family enzyme | CA_C2788 | ND | ND | 2.0 | ND |
| Q97L60 | Predicted lipoprotein, Med/BMP family | CA_C0702 | -3.5 | ND | ND | ND |
| Q97GZ1 | Chemotaxis protein CheW | cheW CA_C2224 | -2.0 | -1.7 | ND | ND |
| Q97GZ5 | Histidine kinase | cheA CA_C2220 | ND | -2.2 | ND | ND |
| Q97GI6 | 2,3,4,5-tetrahydropyridine-2,6-dicarboxylate N-acetyltransferase | dapH CA_C2381 | 1.3 | ND | ND | ND |
| Q97D99 | HAD superfamily hydrolase | CA_C3581 | -1.9 | ND | 4.2 | 8.1 |
| Q97M99 | Protein from nitrogen regulatory protein P-II (GLNB) family, ortholog YAAQ B.subtilis | CA_C0299 | -1.3 | 2.5 | -1.6 | 2.1 |
| Q97MZ9 | ESAT-6-like protein | CA_C0040 | 11.8 | ND | ND | -7.4 |
| P34159 | Uncharacterized protein | CA_C3713 | ND | ND | -6.3 | -9.4 |
| Q97GD5 | HtrA-like serine protease | CA_C2433 | 1.5 | 2.0 | ND | -1.1 |
| **Lipid metabolism** | | | | | | |
| Q97KV0 | Lipase-esterase related protein | CA_C0816 | -6.3 | -1.9 | ND | 2.5 |
| Q97DA9 | 3-hydroxyacyl-[acyl-carrier-protein] dehydratase FabZ | fabZ CA_C3571 | ND | ND | ND | -2.1 |
| Q97DA6 | 3-ketoacyl-acyl carrier protein reductase | fabG CA_C3574 | ND | 1.6 | ND | 1.5 |
| Q97DA7 | 3-oxoacyl-[acyl-carrier-protein] synthase 2 | fabF CA_C3573 | ND | -1.7 | -2.3 | -3.4 |
| P52042 | Acyl-CoA dehydrogenase, short-chain specific | bcd CA_C2711 | ND | -1.5 | ND | -1.9 |
| Q97KP0 | Cyclopropane fatty acid synthase | cfa CA_C0877 | 1.3 | ND | ND | -1.6 |
| Q97DA5 | Malonyl CoA-acyl carrier protein transacylase | fabD CA_C3575 | ND | -1.7 | -1.5 | -2.9 |
| Q97F38 | Pantothenate synthetase (PS) | panC CA_C2915 | ND | -1.2 | ND | ND |
| **Nucleotide metabolism** | | | | | | |
| Q97DA4 | Dioxygenase related to 2-nitropropane dioxygenase | CA_C3576 | ND | -1.7 | ND | -1.4 |
| Q97FR9 | Glutamine synthetase type III | glnA CA_C2658 | -2.0 | -2.0 |  | -1.7 |
| Q97IT9 | Nitroreductase family protein | CA_C1551 | -1.7 | 1.6 | ND | 1.9 |
| Q97IQ7 | Predicted P-loop ATPase | CA_C1583 | -1.4 | -3.5 | ND | -2.2 |
| Q97D53 | 7-cyano-7-deazaguanine synthase | queC CA_C3627 | 1.3 | ND | 1.4 | ND |
| Q97GU0 | Adenine phosphoribosyltransferase (APRT) | apt CA_C2275 | -1.4 | -2.1 | ND | ND |
| Q97EJ9 | Adenylate kinase (AK) | adk CA_C3112 | -1.2 | -1.3 | ND | ND |
| Q97D87 | Adenylosuccinate synthetase (AMPSase) | purA CA_C3593 | ND | -1.6 | -2.2 | -3.1 |
| Q97E92 | Bifunctional protein GlmU [Includes: UDP-N-acetylglucosamine pyrophosphorylase | glmU CA_C3222 | ND | -2.6 | 2.5 | ND |
| Q97HA0 | Bifunctional protein | pyrR CA_C2113 | -1.7 | ND | 2.2 | ND |
| Q97J91 | Bifunctional purine biosynthesis protein PurH [] | purH CA_C1395 |  | -1.5 | -2.6 | -3.7 |
| Q97GN3 | Glucose-1-phosphate thymidylyltransferase | spsI CA_C2333 | 2.4 | 2.5 | ND | 1.5 |
| Q97J90 | Phosphoribosylamine--glycine ligase | purD CA_C1396 | ND | ND | -2.2 | -3.2 |
| Q97J95 | Phosphoribosylaminoimidazole-succinocarboxamide synthase | purC CA_C1391 | -1.2 | -2.4 | 1.8 | ND |
| Q97J93 | Phosphoribosylformylglycinamidine cyclo-ligase ( | purM CA_C1393 | 1.7 | -2.5 | 5.4 | ND |
| Q97E93 | Ribose-phosphate pyrophosphokinase (RPPK) | prs CA_C3221 | ND | ND | -1.6 | -1.6 |
| Q97F73 | Uracil phosphoribosyltransferase | upp CA_C2879 | ND | -1.5 | 1.2 | -1.3 |
| Q97EB1 | Hypoxanthine-guanine phosphoribosyltransferase | hprT CA_C3203 | ND | ND | ND | -1.8 |
| Q97FM8 | Inosine-5'-monophosphate dehydrogenase | guaB CA_C2701 | -1.4 | ND | ND | ND |
| Q97J96 | N5-carboxyaminoimidazole ribonucleotide mutase | purE CA_C1390 | ND | 2.1 | -1.5 | 1.6 |
| **One carbon metabolism** | | | | | | |
| Q97EB3 | Formate--tetrahydrofolate ligase (FHS) | fhs CA_C3201 | -1.2 | -2.0 | -1.8 | -2.9 |
| Q97F85 | S-adenosylmethionine synthase (AdoMet synthase) | metK CA_C2856 | 1.4 | -1.3 | 1.6 | ND |
| Q97GV1 | Serine hydroxymethyltransferase (SHMT) (Serine methylase) | glyA CA_C2264 | ND | ND | -1.9 | -2.5 |
| Q97D82 | Reverse rubrerythrin-1 (revRbr 1) (NADH peroxidase) | rbr3A hsp21 rpr1 CA_C3598 | 1.2 | ND | 3.3 | 1.9 |
| Q97HQ1 | Predicted aldo/keto reductase, YTBE/YVGN B.subtilis ortholog | CA_C1958 | ND | ND | -2.3 | -2.2 |
| Q97EY2 | 2-keto-3-deoxy-6-phosphogluconate aldolase, eda/kdgA | kdgA CA_C2973 | 1.5 | 3.3 | -1.6 | 1.4 |
| Q97GM9 | Phosphomannomutase | CA_C2337 | 1.5 | ND | ND | -3.5 |
| Q97F39 | 3-methyl-2-oxobutanoate hydroxymethyltransferase | panB CA_C2914 | -1.3 |  |  | 3.2 |
| Q97FL3 | Deacethylase/dipeptidase/desuccinylase family of Zn-dependent hydrolases | CA_C2723 | 1.4 | ND | 1.6 | ND |
| Q97EX4 | MinD family ATPase | CA_C2982 | 1.5 | ND | ND | ND |
| Q97LS8 | Peptidase T | pepT CA_C0476 | 1.4 | ND | ND | ND |
| Q97MV3 | Peptide methionine sulfoxide reductase MsrA | msrA CA_C0088 | ND | -1.5 | ND | ND |
| Q97MB9 | Peptidylprolyl isomerase | CA_C0279 | ND | ND | ND | 1.7 |
| Q97F94 | Protein translocase subunit SecA | secA CA_C2846 | ND | ND | -2.6 | -2.3 |
| Q97FT6 | Trigger factor (TF) | tig CA_C2641 | -1.1 | ND | ND | 1.4 |
| P30719 | 10 kDa chaperonin (GroES protein) | groS groES CA_C2704 | ND | ND | ND | 1.3 |
| P30717 | 60 kDa chaperonin (GroEL protein) | groL groEL mopA CA_C2703 | ND | ND | 1.5 | 1.4 |
| Q97ED6 | Cysteine--tRNA ligase | cysS CA_C3177 | ND | -1.5 | ND | -1.6 |
| Q97EF9 | RRNA methylase, YACO B.subtilis ortholog | CA_C3154 | 1.4 | 1.9 | ND | 1.5 |
| Q97GT2 | S-adenosylmethionine:tRNA ribosyltransferase- | queA CA_C2283 | ND | ND | -1.8 | -2.4 |
| Q97N17 | Serine--tRNA ligase 1 ( | serS1 serS CA_C0021 | ND | -1.2 | -1.5 | -1.7 |
| Q97I51 | Translation initiation factor IF-2 | infB CA_C1802 | -1.3 | ND | -1.6 | -1.4 |
| Q97GK5 | Translation initiation factor IF-3 | infC CA_C2361 | 3.2 |  | 3.2 | 2.5 |
| Q97I18 | tRNA-2-methylthio-N(6)-dimethylallyladenosine synthase | miaB CA_C1838 | ND | ND | -2.4 | -1.5 |
| Citric acid cycle |  |  |  |  |  |  |
| O85707 | Thiolase B | thlB | -2.7 | ND | -2.7 | -2.7 |
| Q97KE8 | Aconitase A | citB CA_C0971 | 2.0 | ND | -1.4 | -2.9 |
| Q97KE7 | Isocitrate dehydrogenase | citC CA_C0972 | 3.8 | 1.6 | 1.6 | -1.6 |
| Q97IP4 | Malic enzyme | malS CA_C1596 | -2.7 | -3.6 | ND | ND |
| **Secondary metabolites** | | | | | | |
| Q97LG7 | Pyridoxal 5'-phosphate synthase subunit PdxS | pdxS CA_C0594 | -1.3 | ND | ND | 1.2 |
| Q97LG8 | 6,7-dimethyl-8-ribityllumazine synthase (DMRL synthase) | ribH CA_C0593 | -3.9 | -2.7 | ND | -1.2 |
| **Unknown/other** | | | | | | |
| Q97IC2 | Probable serine/threonine-protein kinase | CA_C1728 | ND | ND | ND | -1.3 |
| Q97GI0 | Uncharacterized protein | CA_C2387 | -1.9 | ND | ND | -1.2 |
| Q97HX6 | Uncharacterized protein | CA_C1880 | -2.0 | -4.4 | 2.8 | ND |
| Q97HY1 | Uncharacterized protein | CA_C1875 | -4.7 | -2.4 | -1.8 | ND |
| Q97K85 | Uncharacterized protein | CA_C1034 | -1.3 | ND | ND | -1.7 |
| Q97KJ9 | Uncharacterized protein | CA_C0920 | ND | ND | ND | -1.3 |
| Q97LR6 | Uncharacterized protein | CA_C0488 | -5.8 | 3.0 | -2.2 | 8.0 |
| Q97M13 | Uncharacterized protein | CA_C0387 | ND | ND | ND | 1.7 |
| Q97M64 | Uncharacterized protein | CA_C0334 | ND | ND | 2.6 | 2.5 |
| Q97MY2 | Uncharacterized protein | CA_C0058 | ND | -5.0 | 3.5 | -1.6 |
| Q97MY3 | Uncharacterized protein | CA_C0057 | ND | -3.2 | 3.8 | ND |
| Q97N05 | Uncharacterized protein | CA_C0034 | -1.6 | 2.4 | ND | 4.0 |
| Q97IL1 | Putative intracellular protease/amidase, ThiJ family | CA_C1629 | ND | ND | 2.2 | 2.5 |
| Q97JJ1 | Uncharacterized conserved protein, YqeY B.subtilis ortholog | CA_C1289 | 1.4 | ND | 1.7 | 2.4 |
| Q97G10 | Uncharacterized protein | CA_C2564 | ND | -1.7 | -1.9 | -3.1 |
| Q97IV0 | Uncharacterized ATP-grasp enzyme | CA_C1540 | 1.4 | ND | 3.7 | 2.1 |
| Q97LC7 | Zinc finger domain | CA_C0635 | -1.2 | ND | -2.4 | -1.7 |
| Q97I96 | UPF0109 protein | CA_C1756 | ND | 2.2 | ND | 2.1 |
| Q97IL7 | Uncharacterized protein, ortholog of Thermotoga | CA_C1623 | -1.2 | 4.2 | ND | 5.0 |
| Q97ID1 | UPF0296 protein | CA_C1717 | ND | 5.3 | -1.6 | 2.6 |
| Q97HX0 | Uncharacterized phage related protein | CA_C1886 | ND | -2.9 | ND | ND |
| Q97HW5 | Uncharacterized protein | CA_C1892 | ND | ND | -2.0 | ND |
| Q97KD3 | Lipoprotein | CA_C0986 | ND | ND | ND | -1.2 |
| Q97IB5 | Predicted kinase related to hydroxyacetone kinase, YLOV ortholog | CA_C1735 | 1.3 | ND | 2.0 | ND |
| Q97L27 | Predicted membrane protein | CA_C0739 | 1.9 | ND | ND | ND |
| Q97CX5 | Hypothetical secreted protein | CA_C3721 | 1.4 | -1.3 | ND | ND |
| Q97CX5 | Hypothetical secreted protein | CA_C3721 | ND | ND | ND | -1.3 |
| Q97I09 | 4-hydroxy-3-methylbut-2-enyl diphosphate reductase | ispH CA_C1847 | 1.1 | ND | ND | -1.2 |

The detailed proteomics information included, Phenyx ID, Quantification ID and Fold changes (iTRAQ ratio; ExpCL vs ExpC, StaCL/StaC, StaC vs ExpC and StaCL vs ExpC) are provided in separate excel file “Supporting information proteomics iTRAQ”.

The detailed metabolites information included cellobiose consumption, cell drybiomass, acetic acid, butyric acid, ethanol, butanol and hydrogen gas are provided in separate excel file “Supporting information metabolites concentrations”.
